# Host-specific adaptation in *Fusarium oxysporum* correlates with distinct accessory chromosome content in human and plant pathogenic strains

**DOI:** 10.1128/mbio.00951-25

**Published:** 2025-06-26

**Authors:** Dilay Hazal Ayhan, Serena Abbondante, Domingo Martínez-Soto, Siyuan Wu, Ricardo Rodriguez-Vargas, Shira Milo, Katherine Rickelton, Vista Sohrab, Shunsuke Kotera, Tsutomu Arie, Michaela Ellen Marshall, Marina Campos Rocha, Sajeet Haridas, Igor V. Grigoriev, Neta Shlezinger, Eric Pearlman, Li-Jun Ma

**Affiliations:** ^1^Biochemistry and Molecular Biology, University of Massachusetts Amherst, Amherst, Massachusetts, USA; 2 Molecular and Cellular Biology, University of Massachusetts Amherst, Amherst, Massachusetts, USA; 3 Graduate School of Natural and Applied Sciences, Acibadem University, Istanbul, Turkey; 4 Physiology and Biophysics and Ophthalmology, University of California, Irvine, California, USA; 5 Department of Microbiology, Centro de Investigación Científica y Educación Superior de Ensenada (CICESE), Ensenada, Baja California, Mexico; 6 Laboratory of Plant Pathology, Graduate School of Agriculture, Tokyo University of Agriculture and Technology (TUAT), Fuchu, Tokyo, Japan; 7 The Robert H. Smith Faculty of Agriculture, Food and Environment, The Hebrew University of Jerusalem, Jerusalem, Israel; 8 US Department of Energy Joint Genome Institute, Lawrence Berkeley National Laboratory, Berkeley, California, USA; Universidad de Cordoba, Cordoba, Spain

**Keywords:** *Fusarium oxysporum*, cross-kingdom fungal pathogen, corneal infection, tomato vascular wilt, accessory chromosomes, comparative genomics

## Abstract

*Fusarium oxysporum* is a cross-kingdom pathogen. While some strains cause disseminated fusariosis and blinding corneal infections in humans, others are responsible for devastating vascular wilt diseases in plants. To better understand the distinct adaptations of *F. oxysporum* to animal or plant hosts, we conducted a comparative phenotypic and genetic analysis of two strains: MRL8996 (isolated from a keratitis patient) and Fol4287 (isolated from a wilted tomato [*Solanum lycopersicum*]). Infection of mouse corneas and tomato plants revealed that, while both strains cause symptoms in both hosts, MRL8996 caused more severe corneal disease in mice, whereas Fol4287 induced more pronounced wilting symptoms in tomato plants. *In vitro* assays using abiotic stress treatments revealed that the human pathogen MRL8996 was better adapted to elevated temperatures, whereas the plant pathogen Fol4287 was more tolerant to osmotic and cell wall stresses. Both strains displayed broad resistance to antifungal treatment, with MRL8996 exhibiting the paradoxical effect of increased tolerance to higher concentrations of the antifungal caspofungin. We identified a set of accessory chromosomes (ACs) that encode genes with different functions and have distinct transposon profiles between MRL8996 and Fol4287. Interestingly, ACs from both genomes also encode proteins with shared functions, such as chromatin remodeling and post-translational protein modifications. Our phenotypic assays and comparative genomics analyses lay the foundation for future studies correlating genotypes with phenotype and for developing targeted antifungals for agricultural and clinical uses.

**IMPORTANCE:**

*Fusarium oxysporum* is a cross-kingdom fungal pathogen that infects both plants and animals. In addition to causing many devastating wilt diseases, this group of organisms was recently recognized by the World Health Organization as a high-priority threat to human health. Climate change has increased the risk of *Fusarium* infections, as *Fusarium* strains are highly adaptable to changing environments. Deciphering fungal adaptation mechanisms is crucial to developing appropriate control strategies. We performed a comparative analysis of *Fusarium* strains using an animal (mouse) and plant (tomato) host and *in vitro* conditions that mimic abiotic stress. We also performed comparative genomics analyses to highlight the genetic differences between human and plant pathogens and correlate their phenotypic and genotypic variations. We uncovered important functional hubs shared by plant and human pathogens, such as chromatin modification, transcriptional regulation, and signal transduction, which could be used to identify novel antifungal targets.

## INTRODUCTION

*Fusarium oxysporum*, a cross-kingdom pathogen, is included in the list of health-threatening fungi by the World Health Organization (1) and is considered to be among the top five most important plant pathogens (2). Corneal infections (keratitis) caused by *Fusarium* pathogens are an important cause of blindness worldwide, resulting in over one million new cases of blindness annually (3, 4). Indeed, *Fusarium* spp. are the most common cause of fungal keratitis in India (5, 6), China (7, 8), South Africa (9), and Brazil (10). *F. oxysporum* was also the cause of the 2005–2006 keratitis outbreak among contact lens wearers in the United States and other temperate regions of the world (11, 12). As a plant pathogen, *F. oxysporum* causes devastating vascular wilt diseases in many economically important crops, including tomato (*Solanum lycopersicum*) (13), cotton (*Gossypium hirsutum*) (14, 15), and banana (*Musa* sp.) (16), and is responsible for billions of dollars in annual yield losses. Disease severity caused by this cross-kingdom pathogen is compounded by the lack of effective drugs, as only a limited number of antifungal agents are available to control eukaryotic fungal pathogens, and most *Fusarium* isolates are notorious for their broad resistance to many of these drugs (17–19).

Host-specific virulence is well recognized among plant pathogenic *F. oxysporum* isolates, as a single *F. oxysporum* species complex (FOSC) member typically infects only one or two plant species and is recognized as a *forma specialis* among plant pathogens. Within the FOSC, over 100 recognized *F. oxysporum formae speciales* infect more than 100 diverse plant hosts (20). We previously identified horizontally inherited accessory chromosomes (ACs) that lack genes associated with housekeeping functions but are enriched for genes related to fungal virulence as determinants of host-specific pathogenicity within the FOSC (21–24). Therefore, ACs (also referred to as pathogenicity chromosomes) encode several functionally validated virulence factors toward plant hosts, such as Secreted in Xylem (*SIX*) effectors (25), transcription factors (26), and kinases (27, 28). The importance of *F. oxysporum* ACs was demonstrated by the finding that a non-pathogenic *F. oxysporum* strain became virulent upon receiving ACs from a pathogenic strain (21, 29, 30).

No specific *forma specialis* has been reported for clinical isolates of *F. oxysporum*. The conventional wisdom was that disseminated fusariosis is the result of opportunistic infection of immunocompromised patients by environmental isolates, and corneal infections occur as a result of ocular trauma or contaminated contact lenses. The finding that a plant pathogenic isolate was able to infect immunosuppressed mice and cause systemic disease supports this notion (31). However, we reported that distinct sets of ACs were reported in the genomes of two clinical FOSC strains (32) and found to lack the genomic signatures that define plant pathogenic ACs, including *SIX* effector genes and some repeat elements commonly present upstream of these genes (33). By contrast, these two clinical isolates share AC regions that are enriched in genes encoding metal ion transporters and cation transporters (32).

Using the well-established murine model of fungal keratitis (4) and the tomato vascular wilt models (25), this study compared the human pathogenic strain MRL8996 isolated from a keratitis patient and the plant pathogenic strain Fol4287 isolated from a diseased tomato to test the hypothesis that distinct ACs contribute to the unique adaptation of fungal strains to animal or plant hosts. Both strains exhibited greater virulence in their respective hosts. Although we observed cross-kingdom virulence, these interactions resulted in milder disease in plants and animals. These observations were supported by *in vitro* studies showing that MRL8996 was better adapted to higher temperatures, while Fol4287 tolerated conditions imposing osmotic or cell wall stress. These phenotypic assays revealed the unique adaptations of human and plant pathogens, providing a platform to dissect their different interactions with diverse hosts. Comparative genomics highlighted distinct genetic elements unique to each strain, offering potential testable hypotheses to correlate phenotypic to genotypic variations between a human and a plant pathogen. We also uncovered important functional hubs in ACs used by both human and plant pathogens, including chromatin modification, transcriptional regulation, and signal transduction, potentially identifying novel antifungal targets.

## RESULTS

### Comparative phenomics reveals host-specific adaptation

We selected two *F. oxysporum* strains, one isolated from a keratitis patient and one from a tomato plant showing wilting symptoms as representative of human and plant pathogens. MRL8996 was originally isolated from an infected cornea of a patient in the 2005–2006 contact lens-associated keratitis outbreak cohort (34). In addition, the genome of this strain has been published (32), and a clinically relevant mouse model of keratitis has been established (35). Phylogenetically, this keratitis strain is grouped with other human pathogenic isolates that cause systemic disease (11, 36). The tomato pathogenic strain Fol4287 was originally isolated from a diseased tomato plant in Spain (36, 37) and has been adopted by the international *Fusarium* research community as a reference strain (21).

#### MRL8996 is more virulent than Fol4287 in infected mouse corneas

**Fig 1:**
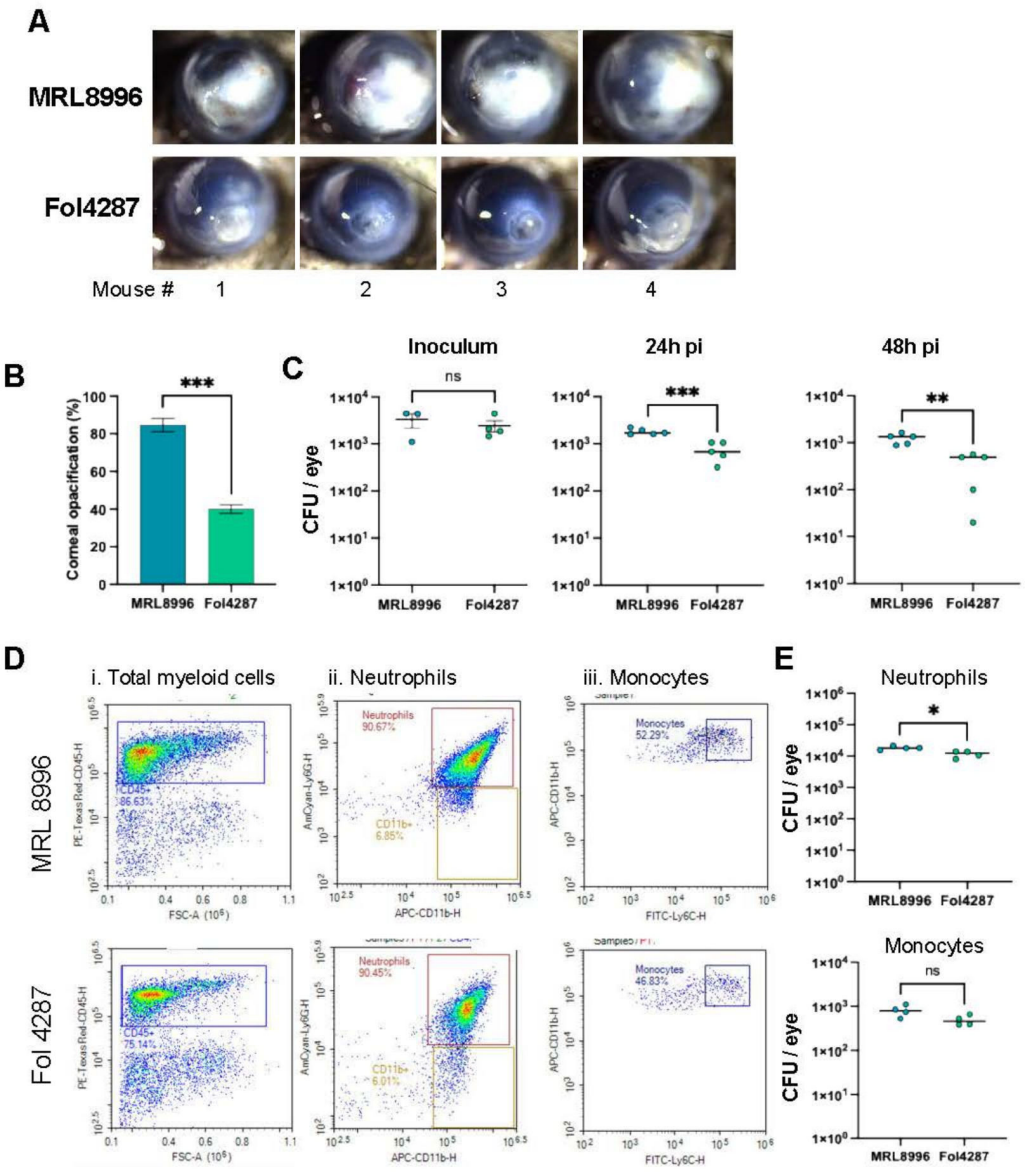
*In vivo* pathogenicity of clinical (MRL8996) and agricultural (Fol4287) *F. oxysporum* strains in infected corneas. (**A**) Representative mouse corneas (*n* = 4) 24 h after intrastromal injection of 5,000 swollen conidia. (**B**) Disease severity, as measured by corneal opacification (38) (***P < 0.001). (**C**) Viable conidia from infected corneas at *t* = 0 (inoculum; 5,000 conidia) and after 24 h, as indicated by the number of colony-forming units (CFUs) (**P < 0.01). (**D**) Infiltrating neutrophils and monocytes identified by flow cytometry. Total cells were isolated from collagenase-digested corneas at 24 h post-infection (hpi) with MRL8996 (top panels) or Fol4287 (bottom panels). Cells were identified using (i) CD45 for total myeloid cells (ii); CD45^+^ Ly6G^+^ and CD11b^+^ to identify neutrophils; and (iii) CD11b^+^ Ly6C^+^ monocytes. (**E**) Quantification of neutrophils (top) and monocytes (bottom) in multiple infected corneas at 24 hpi. Significance was determined by a paired Student’s *t*-test, where *P < 0.05 was considered significant.

To identify potential differences in virulence between Fol4287 and MRL8996 during corneal infection, we infected corneas of C57BL/6 mice with 5,000 *Fusarium* conidia from each strain (inoculum concentrations from 5,000 to 20,000 conidia are shown in Fig. S1). After 24 h, we observed significantly higher corneal opacification in MRL8996-inoculated eyes (Fig. 1A and B), compared to Fol428-inoculated corneas (Fig. 1A and B). We also examined fungal viability following eye infection with either fungal strain and detected significantly more colony-forming units (CFUs) in corneas infected with MRL8996 compared to Fol4287 (Fig. 1C; Fig. S1), indicating that the host was more efficient at containing the infection and possibly killing the Fol4287 strain compared to MRL8996.

The major cause of corneal disease following fungal infection is the recruitment of CD45^+^ myeloid cells, including neutrophils and monocytes, to the corneal stroma (4). To quantify these cells following infection with each *F. oxysporum* strain, we dissected corneas that were infected with MRL8996 or Fol4287, digested them with collagenase, and incubated the infiltrating cells with antibodies against CD45 and CD11b (which recognize total myeloid cells) and Ly6G and Ly6C (which identify neutrophils and monocytes, respectively) (39). Consistent with disease severity, corneas infected with MRL8996 had a higher percentage of CD45^+^ myeloid cells than those infected with Fol4287 (86.6% and 75.1%, respectively) (Fig. 1D–i). However, ~90% of CD45^+^ myeloid cells were Ly6G^+^ neutrophils in corneas infected with each fungal strain (Fig. 1D–ii). Due to the higher percentage of CD45^+^ myeloid cells, we observed significantly more Ly6G^+^/CD11b^+^ neutrophils in corneas infected with the keratitis strain MRL8996 compared to the plant pathogenic strain Fol4287 (Fig. 1E). There was no significant difference in the number of Ly6C^+^/CD11b^+^ monocytes (Fig. 1D–iii and E).

Collectively, these findings indicate that the clinical isolate MRL8996 causes more severe disease and survives better than the plant pathogen Fol4287 in mouse corneas.

##### Fol4287 is more virulent than MRL8996 in infected tomato plants

To examine the relative disease severity caused by these two *F. oxysporum* strains in plants, we inoculated tomato seedlings with MRL8996 or Fol4287 using a well-established wilting disease assay (40–42). Briefly, we soaked cleaned roots of tomato seedlings in a 10^6^ spores/mL suspension for 45 min before gently transplanting the inoculated seedlings in sterilized soil. As a mock control, roots were incubated in sterile distilled water. We used a disease index scale from 1 to 5 to measure disease severity (43), with 1 for healthy plants; 2 for plants showing wilted leaves with chlorotic areas; 3 for plants with necrotic spots; 4 for plants with wilted leaves and whole plants showing chlorosis, areas of necrosis, and defoliation; and 5 for dead plants.

**Fig 2:**
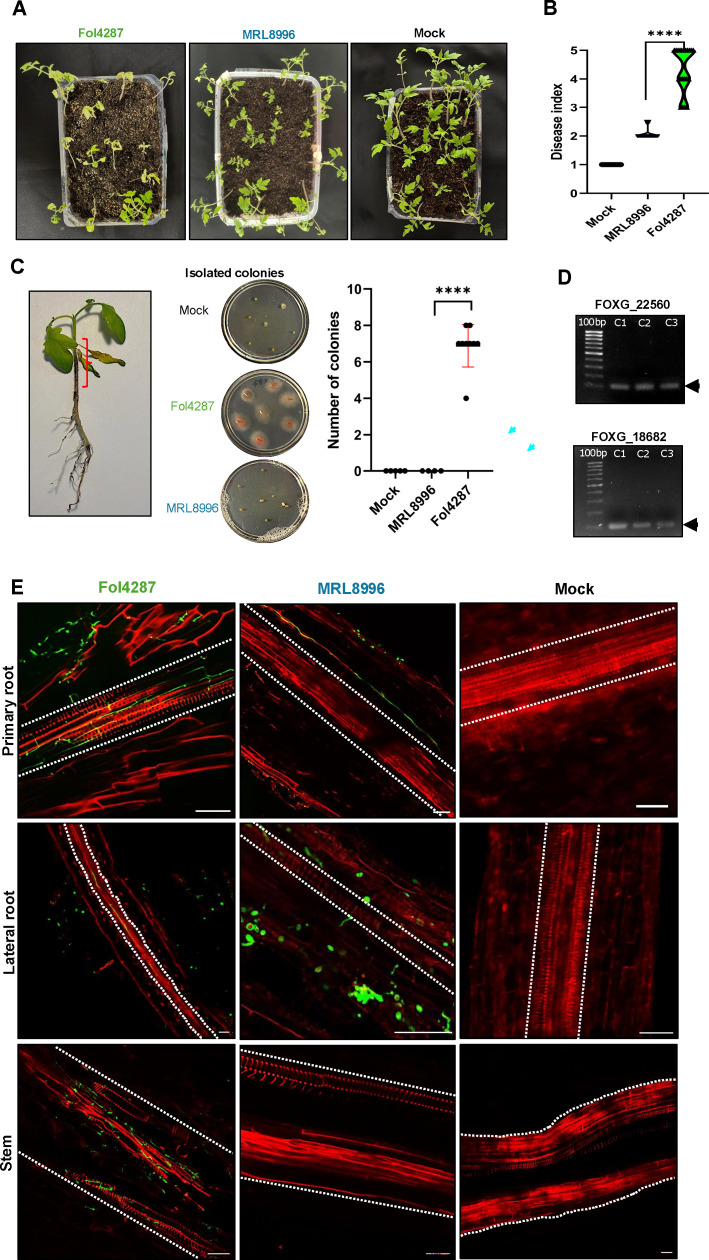
*In vivo* pathogenicity assay of clinical (MRL8996) and agricultural (Fol4287) *F. oxysporum* strains using the tomato vascular disease model. (**A**) Representative photographs of tomato plants inoculated with Fol4287, MRL8996, or water (mock treatment). Ten-day-old tomato seedlings were infected and imaged 10 days later. (**B**) A disease index of 1–5 was used to measure disease severity as described previously (43): 1 for healthy plants; 2 for plants showing wilted leaves with chlorotic areas; 3 for plants with necrotic spots; 4 for plants with wilted leaves and whole plants showing chlorosis, areas of necrosis, and defoliation; and 5 for dead plants. (**C**) Re-isolation of fungal inoculum from inoculated plant stems. The red bracket indicates the area of the plant stem used for fungal isolation. Small slides of the stem were inoculated in Petri dishes with PDA medium. A total of six plants from each treatment were used for this assay. We isolated fungal colonies only from the stems of tomato plants inoculated with Fol4287. (**D**) PCR amplification of 100 bp performed for two specific genes (FOXG_22560 and FOXG_18682) of Fol4287 in three different isolated colonies. (**E**) Tracking the colonization of Fol4287, MRL8996, and mock infection using confocal microscopy. Fungal hyphae (in green) were stained with WGA-Alexa Fluor 488 and detected by excitation at 488 nm and emission at 500–540 nm. Plant tissues (in red) were stained with propidium iodide and detected with excitation at 561 nm and emission at 580–660 nm. Xylem is indicated by the dotted lines. The primary roots, the lateral roots, and the stems are visualized from top to bottom.

At 10 days post-inoculation (dpi), the mock-inoculated seedlings were completely healthy, with a disease index of 1. By contrast, all tomato seedlings infected with the plant pathogen Fol4287 developed severe wilt symptoms, with an average disease index of 4, with wilting leaves showing areas of chlorosis, necrosis, and plant defoliation. Although the keratitis strain MRL8996 also caused chlorosis, the disease symptoms were significantly less pronounced, with an average disease index of 2 (Fig. 2A and B) and only Fol4287 could be re-isolated from inoculated plant stems (Fig. 2C and D). The distinction between these strains was even more pronounced when tracking fungal colonization by confocal microscopy after wheat germ agglutinin (WGA)-Alexa Fluor 488 (green) staining of fungal cell walls and propidium iodide staining (red) of plant cell walls (44). As reported for wilt pathogens (42), the plant pathogenic strain Fol4287 invaded the xylem tissues in both primary and lateral roots at 4 dpi. By contrast, the keratitis strain MRL8996 was restricted to the epidermis and part of the cortex cell layers in both primary and lateral roots (Fig. 2E).

Overall, these findings demonstrate that the plant pathogen caused significantly greater disease severity in tomato seedlings than the clinical pathogen.

##### MRL8996 and Fol4287 exhibit resistance to different abiotic stress conditions

**Fig 3:**
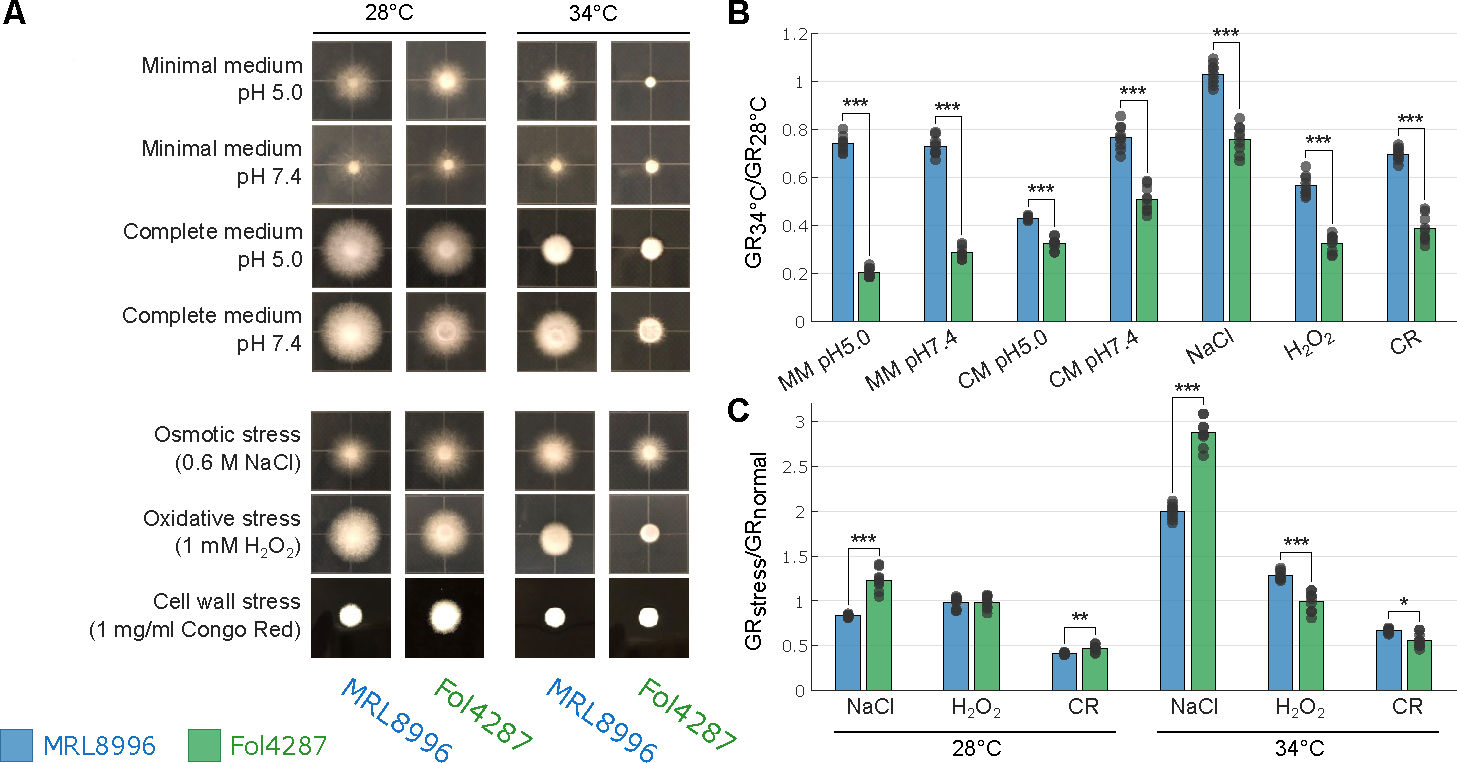
*In vitro* growth of clinical (MRL8996) and agricultural (Fol4287) *F. oxysporum* strains subjected to abiotic stress. (**A**) Colony morphology of MRL8996 and Fol4287 on minimal medium (modified Czapek-Dox agar) pH ~5, minimal medium pH 7.4, complete medium (CM; yeast extract peptone dextrose [YPD] agar), pH ~5, CM pH 7.4, osmotic stress medium (potato dextrose agar [PDA] containing 0.6 M NaCl), oxidative stress medium (PDA with 1 mM hydrogen peroxide [H_2_O_2_]), and cell wall stress medium (YPD with 1 mg/mL Congo Red). The plates were incubated at 28°C or 34°C. The images are representative of three replicates and were taken at 2 days post-inoculation. (**B**) Ratio of the growth rates (GRs) of colonies under the same conditions as in panel **A** at 34°C and 28°C (GR_34°C_/GR_28°C_) as a representation of temperature adaptation. (**C**) Ratio of mean rates of growth under stress conditions (GR_stress_) and in CM (GR_normal_) as a representation of stress tolerance at 28°C and 34°C. Data points indicate each GR value at 34°C normalized to each GR value at 28°C, with each bar showing the mean value. *P < 0.05, **P < 10^−5^; and ***P < 10^−9^, calculated by a two-sample *t*-test.

To explore cross-kingdom adaptation to different environmental conditions, we exposed the two *F. oxysporum* strains to the abiotic stresses of high salinity (0.6 M NaCl), oxidative stress (1 mM H_2_O_2_), and cell wall stress (1 mg/mL Congo Red), or to different temperatures (28°C or 34°C) and pH (5.0 or 7.4). Higher temperatures and pH were chosen since they reflect the conditions of the human cornea (45). We calculated the growth rates (GRs) of each strain at 3 dpi under each condition as the slope of the growth curve (diameter of the colony/dpi). Notably, the keratitis strain MRL8996 formed larger colonies than the plant pathogen Fol4287 in rich and minimal media at both temperatures tested, whereas Fol4287 had a slower GR (Fig. 3A; Table S1; Fig. S2A).

To examine the tolerance of these strains to different stresses, we calculated the ratio of their GRs under two different conditions. For instance, to assess tolerance to different temperatures, we calculated the ratio of the fungal GRs between 34°C and 28°C (GR_34°C_/GR_28°C_). While both strains grew more slowly at the higher temperature, the GR of the plant pathogen Fol4287 was significantly slower at this temperature compared to the keratitis strain MRL8996 under all conditions tested (Fig. 3B and C). At 34°C, lowering the pH from the human physiological pH (7.4) to an acidic pH (5) environment decreased the growth of the keratitis strain by 40%. Our observation that the human pathogenic strain MRL8996 has better tolerance to an elevated temperature of 34°C at pH 7.4 may reflect the adaptation of MRL8996 to the mammalian environment.

Conversely, the plant pathogenic strain Fol4287 exhibited significantly higher tolerance to osmotic stress (0.6 M NaCl) than MRL8996 (Fig. 3C; Fig. S2B). Compared to growth in complete medium alone at 28°C, the growth of MRL8996 in the presence of 0.6 M NaCl decreased by 16.7% relative to the control condition, whereas the growth of strain Fol4287 increased by 22.7%, revealing a significant difference in their response to osmotic stress (*P* < 10^−5^). At 34°C, both strains grew better under higher salinity conditions compared to the complete medium alone. While growth of MRL8996 increased by 99.4% under these conditions, growth of Fol4287 increased by 187.6% (*P* < 10^−9^). Therefore, the plant pathogenic strain Fol4287 is better adapted to higher salinity conditions than MRL8996 at both temperatures (Fig. 3C; Fig. S2B).

Surprisingly, 1 mM H_2_O_2_ treatment (to induce oxidative stress) did not affect the GRs of either strain at 28°C. However, the human pathogenic strain MRL8996 exhibited resistance to oxidative stress, with a 28.4% increase in growth at 34°C compared to samples grown in complete medium alone. Further, cell wall stress imposed by the addition of 1 mg/mL Congo Red to the medium inhibited the growth of both strains. At 28°C, compared to growth in the YPD medium alone, the GRs of MRL8996 and Fol4287 were significantly lower (by 58.8% and 52.9%, respectively). At 34°C, the GR was reduced by 33.3% for MRL8996 and 43.8% for Fol4287 compared to the control.

We repeated *in vitro* phenotyping of the two reported strains and added one additional plant pathogenic isolate Fo5176 (infecting *Arabidopsis thaliana*) and one additional clinical isolate NRRL 32931 (isolated from a leukemia patient) (Fig. S2; Table S1) and observed consistent results. While GRs were reduced for all four strains at 34°C compared to 28°C, the reduction of the two plant strains was significantly greater than the two clinical isolates. MRL8996 exhibited the highest tolerance to heat compared with Fo5176. The clinical isolate NRRL 32931 grew significantly better at physiological pH (7.4) compared to the acidic pH (5) for both minimal media and complete media (Fig. S2B). Also consistent with the previous findings, the plant isolates show higher resistance to high salinity in both temperatures compared to the two human clinical isolates. Fo5176 surprisingly shows high resistance to salinity and cell wall stress under both temperatures, with the difference more pronounced at 34°C (Fig. S2C).

Taken together, these findings indicate that the clinical isolate MRL8996 is better adapted to the physical conditions of the animal host, such as elevated temperature, whereas the plant pathogenic strain Fol4287 is more tolerant to increased salinity. These findings reflect a complex, interconnected regulatory relationship between the physiological adaptation of the fungus and fungal–host interactions.

##### MRL8998, but not Fol4287, exhibits tolerance to high caspofungin concentrations

Caspofungin acetate (CFA) is an antifungal agent which targets 1,3-β-glucan synthase that mediates biosynthesis of one of the main components of the fungal wall (46, 47). However, different fungi show diverse responses to CFA, as clinical isolates of *Candida albicans* and *Aspergillus fumigatus* are sensitive to CFA (48), whereas some less common fungal human pathogens, including *Fusarium* spp., are resistant to clinically relevant levels of CFA (49).

**Fig 4:**
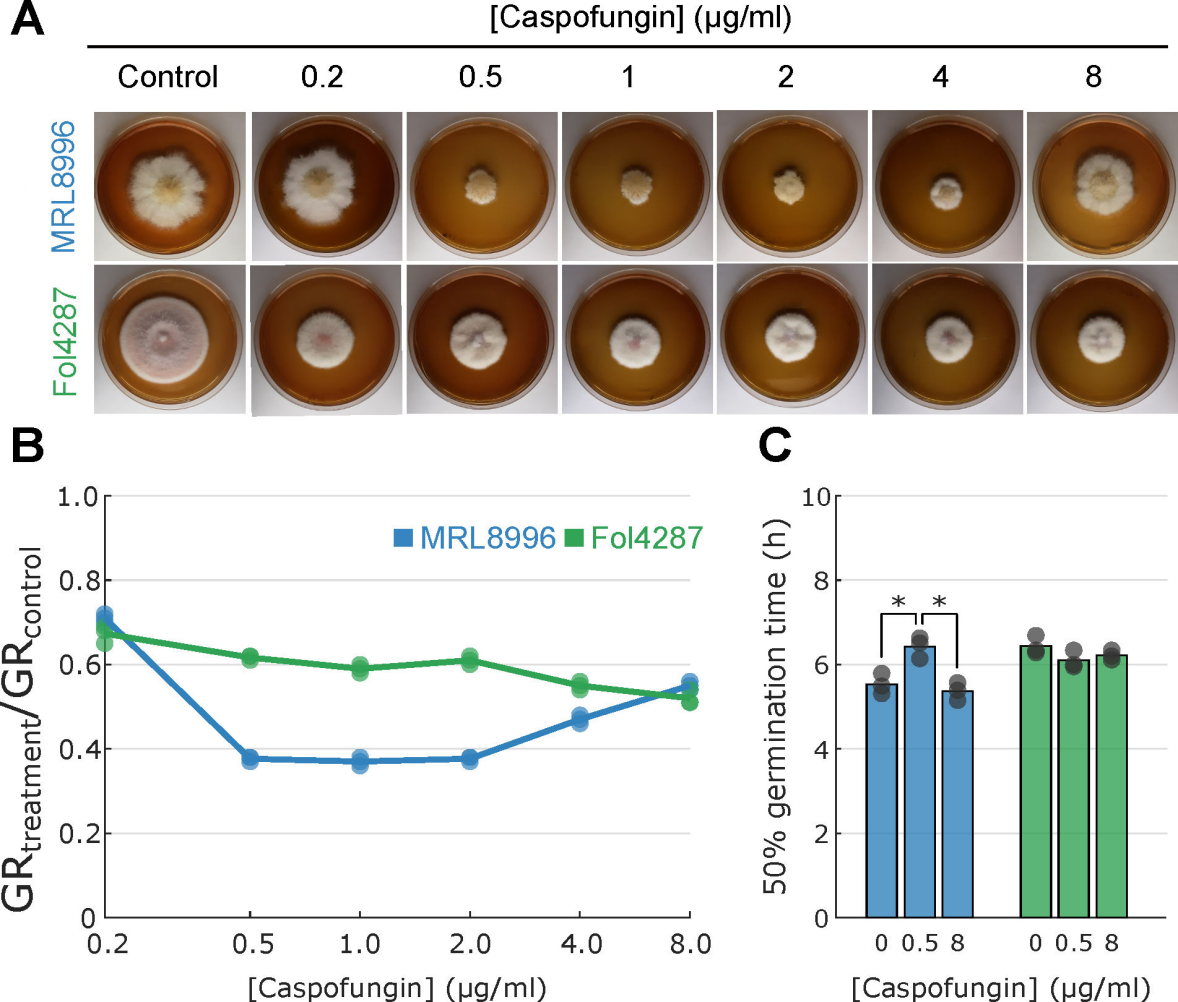
*F. oxysporum* keratitis strain MRL8896 exhibits a paradoxical effect to caspofungin. (**A**) Representative images of radial fungal growth in the presence of caspofungin. (**B**) Ratio of growth rates (GRs) for colonies in the presence of caspofungin (treatment) and in the absence of caspofungin (control). (**C**) Effect of caspofungin concentration on germination time. For each strain, 1 × 10^4^ conidia were inoculated onto glass coverslips containing 200 µL of liquid PDA medium and incubated at 28°C for 24 h. Two hundred conidia were counted, and the percent germination was calculated at 0, 2, 4, 6, 8, and 12 h. The time when 50% of the spores germinated was calculated based on the closest data point in a log-logistic regression.

In the current study, the keratitis strain MRL8998 exhibited enhanced tolerance to high caspofungin concentrations compared with Fol4287 (Fig. 4A; Fig. S3). The GR of the plant pathogen Fol4287 decreased from 80% to 70% with an increase in caspofungin concentration from 0.2 to 8 µg/mL compared to growth in the absence of caspofungin, showing a strong tolerance to caspofungin without a clear dose-dependent response. In contrast, the GR of the keratitis strain MRL8996 dropped by 50% under 2 µg/mL caspofungin, with a further reduction to a 35% decrease at 8 µg/mL, relative to the control (Fig. 4B). This paradoxical caspofungin effect was also observed when measuring the time required to reach 50% conidial germination (Fig. 4C). While there was no significant difference in the time needed for the plant pathogen Fol4287 to reach a 50% germination rate in the presence of caspofungin, the human keratitis strain required a significantly longer time to reach 50% germination under 0.5 µg/mL caspofungin treatment compared to the no caspofungin control or 8 µg/mL caspofungin treatment. Taken together, these findings indicate the different responses toward the antifungal caspofungin.

### Comparative genomics reveals strain-specific ACs with distinct transposon profiles

**Fig 5:**
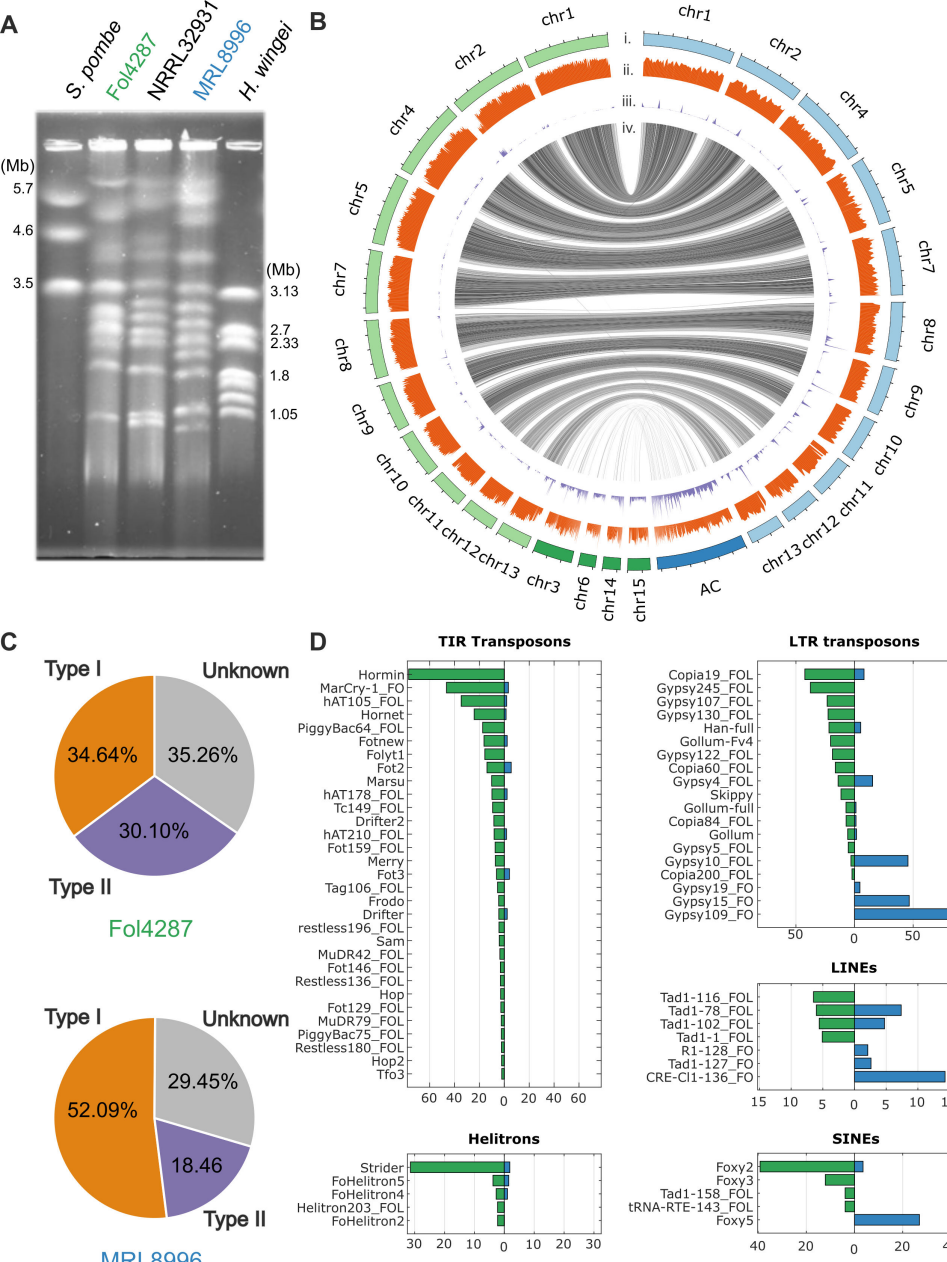
Compartmentalized genomes of the clinical (MRL8996) and agricultural (Fol4287) *F. oxysporum* strains. (**A**) Contour-clamped homogeneous electric field (CHEF) gel of small accessory chromosomes (ACs) of the agricultural isolate Fol4287, the control clinical isolate NRRL32931, and the clinical isolate MRL8996. *Schizosaccharomyces pombe* (left) and *Hansenula wingei* (right) were used as markers. The band sizes are shown in Mb. ACs are typically below 2 Mb in size, except for two known ACs, chr3 and chr6 in Fol4287, which are much larger due to recent segmental duplications (21). (**B**) Whole-genome comparison between MRL8996 and Fol4287 revealed 11 homologous core chromosomes (light green and light blue, respectively) and accessory sequences (dark green and dark blue, respectively). (i) The accessory sequences typically displayed low gene density (ii) and high repetitive sequence composition. (iii) The syntenic alignment between MRL8996 and Fol4287 using nucmer (iv) indicates the accessory regions lacking synteny between MRL8996 and Fol4287. (**C**) Distribution of all identified transposable elements in the Fol4287 (upper panel) and MRL8996 (lower panel) genomes, consisting of class I retrotransposons, class II DNA transposons, and unknown transposable elements. (**D**) Transposon abundance in the Fol4287 and MRL8996 genomes. Class I retrotransposons include LTRs (long terminal repeats), LINEs (long interspersed nuclear elements), and SINEs (short interspersed nuclear elements). Class II DNA transposons are classified into TIRs (terminal inverted repeats) and Helitrons.

We used a contour-clamped homogeneous electric field (CHEF) gel to observe the diversity of karyotypes among *F. oxysporum* genomes and confirmed the presence of ACs in Fol4287 (21, 50), MRL8996, and NRRL 32931 (32) (Fig. 5A). Comparative genomics confirmed the conservation of core chromosomes (CCs) (Fig. 5B; Table S2) and revealed three small CCs (chromosomes 11–13) that were less conserved, with an average of 94.0% sequence identity over 35.1% coverage. These findings support the three-speed evolutionary hypothesis (51). The conservation of CCs enabled us to identify ACs and sequences unique to the plant and human strains. As highlighted in darker blue and darker green boxes in Fig. 5B, the genomes of MRL8996 and Fol4287 contained 6.4 and 9.8 Mb of accessory sequences corresponding to 12.8% and 18.7% of the total genome, respectively. Our comparative analysis also revealed overall conservation of the mitochondrial genome (99.0%) and highlighted a divergent sequence around open reading frame 2285 (ORF2285) (Fig. S4), a known variable mitochondrial region in the genus *Fusarium* (52).

The repeat contents in the Fol4287 and MRL8996 genomes were similar, with 6.6% and 5.4% values, respectively (Files S1 and S2). Transposable elements (TEs), a signature of accessory sequences (53), were enriched in the ACs of both genomes (27.2% and 23.8%, respectively) (Table S3), but with distinct transposon profiles. In the Fol4287 genome, approximately one-third of identified TEs were type I retrotransposons, and another third was type II DNA transposons. In the MRL8896 genome, more than half were identified as type I and less than 20% as type II TEs (Fig. 5C).

Some class I retrotransposons are conserved within the genus of *Fusarium* (21). Similarly, the long terminal repeat (LTR) transposon *Copia* was present in both the Fol4287 and MRL8996 genomes (Fig. 5D; Table S4). However, some LTR elements with the highest copy numbers in MRL8996, such as several *Gypsy* elements, were not present in Fol4287. Similarly, the most abundant long interspersed nuclear element (LINE) transposon in MRL8996, the *Cnl1* CRE-type non-LTR retrotransposon, was not present in Fol4287. Of the three short interspersed nuclear element (SINE) transposon families, the *Foxy2* family was the most abundant in Fol4287, while the *Foxy5* family was the most abundant in MRL8996. Conversely, Fol4287 contained unique class II DNA transposons (Fig. 5D; Table S4).

The most abundant DNA TE in Fol4287, *Hormin* (a miniature *Hornet* element), was absent from the MRL8996 genome. Miniature impala elements (MIMPs) (33) were not present in MRL8996. Similarly, Fol4287 contained significantly more Helitrons (54) compared to MRL8996. There were also many unclassified repeat elements that were not shared between the Fol4287 and MRL8996 genomes (Fig. S5). The distinction between different TE profiles is important, as class I and class II TEs play different roles in the genome dynamics of MRL8996 and Fol4287.

### Strain-specific ACs contribute to distinct functional adaptations

**Fig 6:**
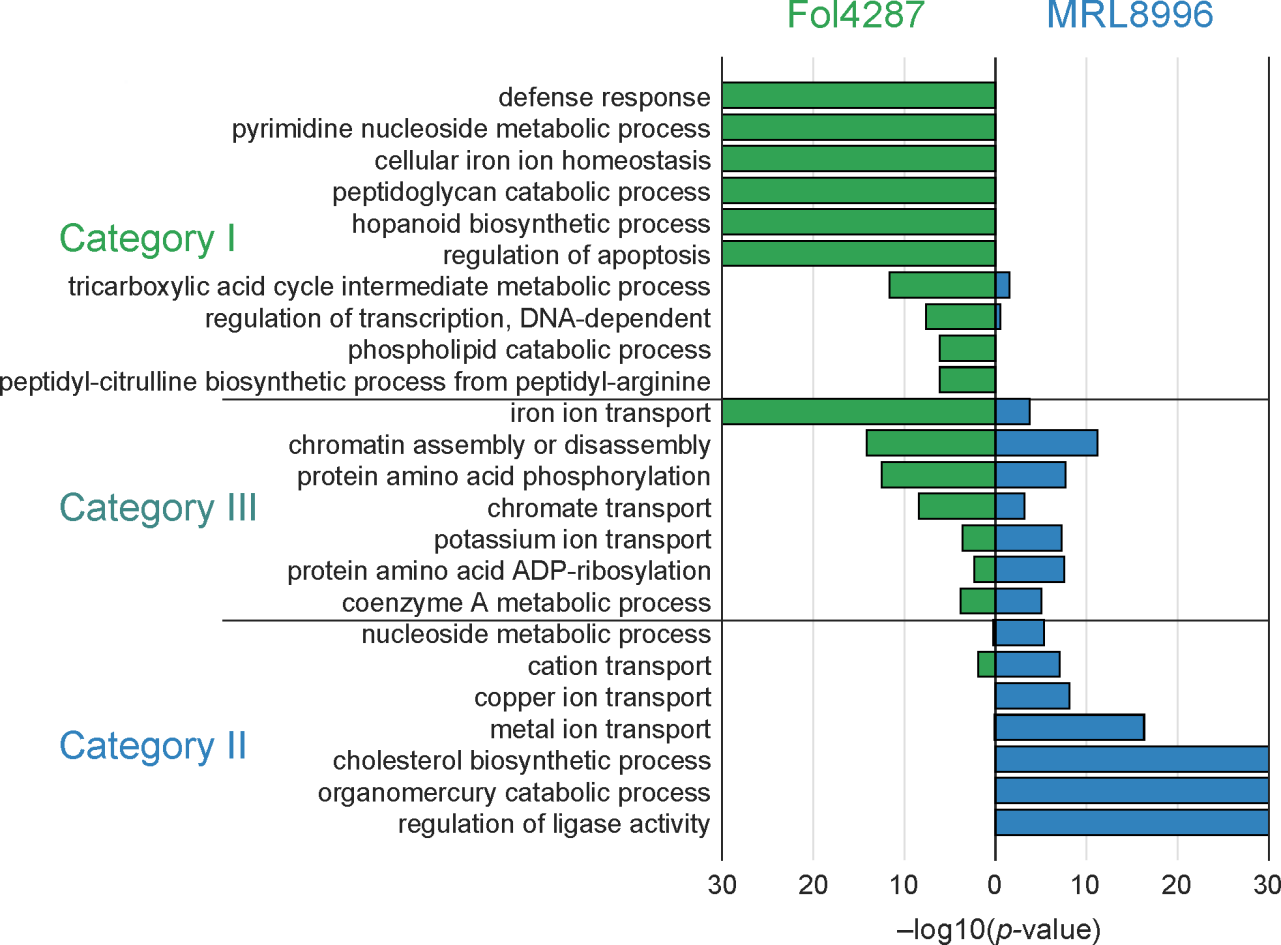
Enriched gene ontology (GO) terms among accessory genes of the clinical (MRL8996) and agricultural (Fol4287) *F. oxysporum* isolates. Enriched GO terms harbored by the ACs from Fol4287 (green) and MRL8996 (blue). Category I represents GO terms enriched in Fol4287 ACs. Category II represents GO terms enriched in the ACs of both genomes. Category III represents GO terms enriched in MRL8996 ACs. For graphical representation, a *P* value = 0 is shown as −log10(*P* value) = 30.

We annotated the genome of MRL8996 using the Joint Genome Institute annotation pipeline (55) and assigned gene ontology (GO) terms to genes in both genomes using the Mycocosm GO annotation pipeline (Table 1). Among the 16,631 and 20,925 predicted protein-coding genes in the MRL8996 and Fol4287 genomes, respectively, we identified 2,017 (12.1%) and 3,890 (18.6%) genes located on their respective ACs. GO functional enrichments (*P* value < 10^−3^) revealed some functional categories shared by both pathogens (Fig. 6 category III), in addition to strain-specific functions (Fig. 6 categories I and II, Tables S5 and S6).

**TABLE 1:** Comparative annotation of the Fol4287 and MRL8996 genomes

Parameter	Fol4287	MRL8996
Total genes	20,925	16,631
Genes with GO annotation	9,783	7,958
Total AC genes	3,890	2,017
AC genes with GO annotation	1,788	542

#### Functional groups enriched in both genomes due to different sets of AC genes

- Chromatin assembly or disassembly: the most significantly enriched functional term in both genomes was chromatin assembly or disassembly (GO:0006333), with *P =* 6 × 10^−12^ for MRL8996 AC genes and *P =* 7 × 10^−15^ for Fol4287 AC genes. Most genes under this annotation encode CHROMO domain-containing proteins that bind to the H3K9 di/trimethyl modification on histone H3, a hallmark of heterochromatic regions (56, 57) that are typically involved in the assembly or remodeling of chromatin and associated proteins or RNAs (58). Phylogenetic analysis grouped these genes into three clades (Fig. S6). The first clade shares homologs of CHROMO domain-containing protein 2 (Chp2) from fission yeast (*Schizosaccharomyces pombe*). The second clade encodes homologs of putative Heterochromatin protein 1 (HP1) (A0A0C4BKY0) based on AlphaFold prediction (59). The third clade includes genes encoding zinc finger C2H2-type domain-containing proteins. Notably, Chp2 and HP1 both interact with chromatin in DNA–protein complexes (60), and functioning as a key regulator coordinating eukaryotic chromatin compaction, HP1 can bind to heterochromatin marks and recruits other factors to promote heterochromatin formation (61). *F. oxysporum* ACs are primarily composed of facultative heterochromatin (51). The equal expansion in this protein family suggests that establishing the ability to effectively open or close AC regions, either to exploit transcriptional networks or to allow genome maintenance activities, likely plays a crucial role in the functional regulation of *F. oxysporum* ACs.
- Post-translational modifications: the other significantly enriched term (*P* = 1.95 × 10^−8^ for MRL8996 AC genes and *P =* 3 × 10^−13^ for Fol4287 AC genes) was protein phosphorylation (GO:0006468), an important cellular regulatory mechanism through kinases. This result was expected, as we previously identified a positive correlation between the total number of protein kinases encoded in a genome and the size of the proteome of an organism (62). However, each genome has its own distinct set of encoded proteins. For instance, the major AC-enriched kinases in MRL8996 are halotolerance protein 4-like (HAL4) serine/threonine kinases (which regulate ion transporters), calmodulin-dependent protein kinase 2 (which is involved in energy balance and perhaps mobile DNA repair activity), and cell division cycle-like kinases (which function in chromatin remodeling and DNA metabolism/repair). The kinases encoded by the AC of Fol4287 include the second copy of a TOR kinase paralog (a top regulator that dictates cellular stress responses) (62); choline kinase (a conserved APH superfamily member involved in antibiotic resistance); a TOMM system kinase/cyclase fusion protein; and the serine/threonine kinase protein kinase B (involved in stress responses and signal transduction).

In addition to phosphorylation, the other shared GO term involved in the post-translational modification of proteins is protein ADP-ribosylation (GO:0006471) (*P =* 2.99 × 10^−8^ for MRL8996 AC genes and *P =* 5 × 10^−3^ for Fol4287 AC genes). Several transporter genes, including a chromate transporter gene (GO:0015703), genes enriched in the terms potassium ion transport and iron ion transport, and a hydroxymethylglutaryl-CoA reductase gene (GO:0015936), the rate-limiting enzyme for ergosterol biosynthesis, were also enriched in both genomes (Fig. 5C; Tables S5 and S6).

##### Fol4287 AC genes are uniquely enriched for defense responses and signaling

Among the best characterized Fol4287 AC genes are *SIX* effector genes, encoding *bona fide* fungal virulence factors (23, 63, 64) that are directly involved in fungal–plant interactions. Many genes identified in this study were reported previously. In addition, this study revealed a significant enrichment of Fol4287 AC genes in the GO term defense response (GO:0006952) compared to the core genes and the AC genes from MRL8896 (Fig. 6 category I, Table S6). The AC genes in this group include two chitinase II genes and one patatin-like phospholipase gene involved in the manipulation of plant resistance proteins (65).

##### MRL8996 AC genes are uniquely enriched for metal ion transport and catabolism

Whereas MRL8996 ACs lack *SIX* effector genes and genes encoding plant cell wall–degrading enzymes, unlike plant pathogenic *F. oxysporum* strains (32), other effectors are also detected. Also, MRL8996 ACs are uniquely enriched in genes involved in the transport and catabolism of metal ions such as copper, zinc, magnesium (GO:0006825 and GO:0030001), and mercury (GO:0046413) (Fig. 6 category II, Table S5). Nine genes are specifically involved in copper ion transport, with seven Ctr-type copper transporter paralogs and two copper-exporting P-type ATPase (CopA) paralogs encoding proteins containing multiple copies of the copper chaperone (CopZ) domain. Other genes included in these categories encode a CorA-like magnesium/zinc transporter, with multiple copies of ankyrin repeats, and a Zrt-like, Irt1-like protein (ZIP)-type zinc ion transporter. Most of these genes are present in the genome of another clinical isolate, NRRL32931 (25 out of 33 genes). One of these shared genes encodes a mammalian ceruloplasmin homolog with two multicopper oxidase domains. Homologous sequences were previously detected among a few other opportunistic fungal pathogens and bacteria isolated from extreme environments, but not in plant pathogenic *Fusarium* species (32), suggesting that a host-specific gene network evolved in the human pathogens. Another GO term that was exclusively enriched in the MRL8996 ACs is the regulation of ligase activity (GO:0051340).

Taken together, our results provide strong evidence that different ACs acquired by a plant or a human pathogen perform shared functions that are essential for chromatin modifications, transcriptional regulation, and post-translational modification of proteins. These essential regulatory mechanisms, which are involved in environmental sensing and cellular signal transduction, may hold the key to understanding the crosstalk between the core and AC genomic regions. However, the strain-specific AC gene repertoires are uniquely enriched for defense responses and signaling in the plant pathogen and metal ion transport and catabolism in the human pathogen. We propose that these unique adaptations are important for fungal survival in different environments and hosts.

## DISCUSSION

The FOSC, a group of cross-kingdom fungal pathogens, includes both plant and animal pathogens. Our *in vivo* assays using animal and plant hosts confirmed the host specificity of the keratitis strain MRL8996 and the tomato pathogen Fol4287, as corneal infection with the keratitis strain resulted in significantly more corneal ulceration, while the plant pathogen caused significantly more wilt symptoms among all inoculated tomato seedlings. Consistent with host-specific adaptations, the keratitis strain MRL8996 grew significantly better at elevated temperatures, whereas the tomato pathogen Fol4287 exhibited more tolerance to osmotic and cell wall stress. As a line of fungal adaptation, a human pathogen must overcome the host defenses associated with mammalian endothermy and homeothermy, while a plant pathogen must handle different stresses (66, 67). Our findings reveal interconnected fungal responses toward biotic stress from plant and animal hosts and abiotic stress in distinct environments. The distinct phenotypes observed in our *in vivo* and *in vitro* assays, together with the identification of unique ACs in each genome, lay the foundation for dissecting cross-kingdom fungal pathogenicity using this comparative model.

The widespread occurrence of the caspofungin paradoxical effect underscores the complexity of antifungal drug responses in filamentous fungi and provides an early warning about the potential complexity of treating infectious diseases caused by *Fusarium* species (68). In the current study, we detected the caspofungin paradoxical effect, but only for the keratitis strain MRL8996. Mechanisms underlying this phenomenon likely involve specific drug–target interactions for each strain (69), fungal stress responses (70), and cellular signaling pathways (68, 71–75). Future studies will identify underlying mechanisms that may lead to strategies to overcome the paradoxical effect and improve the efficacy of antifungal therapies.

In addition, we observed severe disease phenotypes from *Fusarium* keratitis compared to other fungal infections. For instance, corneal infection with *A. fumigatus* requires 40,000 conidia as the inoculum, which still causes severe corneal disease (76). When we used the same titer of *F. oxysporum* conidia, almost all animals developed corneal ulceration and perforation within 24 h. Therefore, we lowered the inoculum to 5,000 conidia. This hypervirulence may contribute to *Fusarium* species being the leading cause of blindness among fungal keratitis patients (3, 5).

A unique advantage of our comparative system is the compartmentalized genomic structures for both pathogens, allowing us to characterize two distinct sets of ACs that revealed distinct transposon profiles: while DNA transposons dominate Fol4287 ACs, retrotransposons, especially LINE transposons, are highly abundant among MRL8996 ACs. Intriguingly, ACs from both genomes also encode proteins with shared functions, including chromatin assembly/disassembly, protein phosphorylation, and transcriptional regulation, which support our previous findings (26, 62). Our observation that there is shared enrichment of genes involved in chromatin assembly and disassembly points to the importance of chromatin remodeling regardless of host-specific functions. Identifying master regulators of the crosstalk between ACs and core components, especially those regulating the activities of both plant and animal pathogens, should uncover new targets for control of this cross-kingdom pathogen.

In summary, this is the first study documenting similarities and differences in the genotypes and phenotypes of two closely related pathogens: one infecting humans and one infecting plants. Our *in vivo* and *in vitro* assays allowed us to examine strain adaptation under different feasible environmental stress conditions. In addition to host-specific virulence, we also observed cross-kingdom virulence, as the human pathogen also colonized plant roots and the plant pathogenic strain infected corneas, although both resulted in milder disease symptoms. More in-depth research is needed on the molecular mechanisms underlying species divergence and adaptation among this group of pathogens.

## MATERIALS AND METHODS

### Fungal strains

The genome of *F. oxysporum* Fol4287 was first sequenced in 2010 (21), and an improved genome assembly was subsequently produced in 2018 (50). The strain is deposited in multiple public strain repositories, including the Fungal Genetics Stock Center (FGSC 9935), NCAUR/USDA (NRRL 34936), and CBS-KNAW (CBS 123668). The keratitis strain *F. oxysporum* MRL8996 was originally isolated in 2006 from the cornea of a patient with the contact lens-associated multistate outbreak fungal keratitis at Cleveland Clinic Foundation in Ohio, USA (34). The strain is available at NCAUR/USDA (NRRL 47514). MRL8996 is grouped with other human pathogenic isolates belonging to clade FOSC 3a. The genome assembly for MRL8996 was produced using the same sequencing technologies and computational strategies used to assemble the Fol4287 genome to facilitate effective comparative analysis (32).

### Fungal growth conditions

The conidia of the fungal strains were stored at −80°C in an ultra-freezer in 25–50% (vol/vol) glycerol for long-term storage and propagated from stocks in liquid potato dextrose broth ( BD Difco, USA) or potato sucrose broth (PSB; 25% [wt/vol] boiled potato and 0.5% (wt/vol) sucrose) for at least 4 days in a shaking incubator at 28°C. The conidia were collected by filtering the liquid fungal cultures through two layers of Miracloth (EMD Millipore). The filtrate was centrifuged at 3,700 × *g* for 5 min and the spores were resuspended in sterile water to the desired concentration. The spore titers were determined using a hemocytometer.

### Mouse corneal infection

Fungal conidia were harvested from Sabouraud Dextrose Agar (SDA) medium and resuspended in phosphate-buffered saline (PBS; pH 7.0). Mice were anesthetized by intraperitoneal injection with ketamine/xylazine. A 30-gauge needle was used to make a pocket in the corneal stroma, after which a 33-gauge Hamilton syringe was inserted, and 5 × 10^3^ conidia in 2 µL PBS was injected into the corneal stroma. Corneal opacity was photographed using a high-resolution stereo fluorescence MZFLIII microscope (Leica Microsystems) and a Spot RT Slider KE camera (Diagnostics Instruments). All images were captured using SpotCam software (RT Slider KE; Diagnostics Instruments). Corneal opacity was quantified using ImageJ software. The experiments used five mice per group and were repeated four times.

### CFUs from *Fusarium*-infected corneas

Infected whole eyes were homogenized in 1 mL of sterile PBS using a Retsch Mixer Mill MM300 (Qiagen, Valencia, CA) at 33 Hz for 4 min. Tenfold dilutions were made, and the samples were plated on SDA plates and incubated at 34°C for 48 h. CFUs were determined by direct counting.

### Flow cytometry

Infected corneas were dissected, the vascularized iris was removed, and the corneas were incubated in 500 µL collagenase type I (Sigma-Aldrich) at a titer of 82 U per cornea for 1–2 h at 37°C. Cells were resuspended in 200 µL FACS buffer containing 4 µg/cornea Fc blocking Ab (anti-mouse CD16/32; eBioscience) on ice for 10 min, followed by incubation with anti-mouse antibodies against CD45, Ly6G, CD11b, or Ly6C, all from BioLegend. Total cells were quantified using an ACEA Novocyte flow cytometer.

### Phenotyping of isolates

Yeast extract peptone dextrose (YPD) plates were used as complete medium, and modified Czapek-Dox plates were used as minimal medium. The pH levels of both media were adjusted to pH 7.4 using 6.5% (wt/vol) 0.1 M citric acid and 43.5% (wt/vol) 0.2 M Na_2_HPO_4_. YPD plates containing 0.6 M NaCl, 1 mM H_2_O_2_, and 1 mg/mL Congo Red were prepared for osmotic, oxidative, and cell wall stress treatments, respectively. Each isolate (Fol4387, MRL8996, NRRL 32931, and Fo5176) was grown in PSB for 4 days at 28°C, and the conidia were filtered as described above. Plates containing the same medium were inoculated with 2 µL of spore suspensions at concentrations of 5 × 10^6^, 5 × 10^5^, or 5 × 10^4^ conidia/mL, each in three replicates. The plates were incubated at 28°C or 34°C for 3 days and photographed once a day. The experiments were repeated two times.

### Statistical analysis of GRs

The GRs of each replicate and dilution were calculated as the slope of the 3 day growth curve. Five-way ANOVA with a linear model was performed for the following groups: strain, temperature, medium, dilution, and replicate, followed by a multi-comparison with strains, temperatures, and media groups to identify significant differences using MATLAB. To quantify adaptation to different temperatures, the GRs of cultures with an initial inoculum of 1 × 10^3^ conidia were measured in triplicate. The values of the three replicates incubated at 34°C were normalized to the values of the three replicates incubated at 28°C (total of nine values). A two-way Student’s *t*-test was performed between Fol4287 and MRL8996 values. Similarly, to quantify the effects of stress treatment, the values of the three replicates of samples in medium containing different stress components were normalized to the values of the three replicates in control YPD medium (total of nine values), followed by a two-way Student’s *t*-test between Fol4287 and MRL8996 in MATLAB.

### Caspofungin sensitivity assay

Both strains were maintained in potato dextrose agar (PDA) medium. For solid medium, 2% (wt/vol) agar was added. The GR was determined by spotting 1 × 10^4^ conidia in the center of a 90 mm Petri plate containing 20 mL of solid PDA medium and incubating the plates at 28°C. The diameter was scored at 24 h intervals until 5 days (96 h) of incubation with three biological replicates. To assess the strains' germination kinetics, 1 × 10^4^ conidia of each strain were inoculated onto glass coverslips containing 200 µL liquid PDA medium, and the samples were incubated at 28°C. A conidiospore was counted as germinated if it possessed a germ tube, which was readily detectable as a small protuberance on the spherical spore surface. Two hundred conidia were counted in each experiment. The time required for 50% of conidia to initiate germ tube formation was determined.

### Plant infection assay

Seeds of tomato (*S. lycopersicum*) cv. M82 was maintained in the dark at 4°C for 3 days. The seeds were surface sterilized in 70% (vol/vol) ethanol for 5 min and washed with 2.7% (wt/vol) sodium hypochlorite (NaClO) for 5 min. After removing the NaClO solution, the seeds were rinsed with sterile distilled water (SDW) three times. The seeds were then sown in pots filled with autoclaved soil (Promix BX) and watered with SDW. The soil was gently removed from the roots of 10-day-old tomato seedlings by rinsing them with abundant distilled water and SDW while avoiding root tissue damage. The roots of the seedlings were inoculated by dipping them into the respective human or plant pathogenic *Fusarium* spore suspension (10^6^ spores/mL) or in SDW as a mock infection control for 45 min before replanting the infected seedlings in soil. After infection, all seedlings were maintained under controlled conditions in a growth chamber at 28°C under a 14-h-light/10-h-dark photoperiod.

### Visualization of *F. oxysporum* colonizing plant tissue using confocal microscopy

Plant stems and roots were stained following a published protocol using WGA coupled to the fluorophore Alexa Fluor 488 (WGA-Alexa Fluor 488) and propidium iodide (PI) (42, 44). Stained samples were visualized using an Olympus FluoView FV1000 confocal microscope (Tokyo, Japan), and images were acquired with photomultiplier tube detectors Hamamatsu R7862. Processed samples are sequentially imaged as WGA-Alexa Fluor 488 is detected at an excitation wavelength of 488 nm and an emission range of 500–540 nm, and PI is detected at an excitation of 561 nm and an emission range of 580–660 nm. The experiments were repeated two times.

### Isolation and molecular verification of *F. oxysporum* passaging through plant vascular tissues using selective culture and strain-specific PCR

Tomato stems were harvested after 16 dpi and washed with abundant SDW. Under the laminar flow hood, stems were dipped in 10% NaClO for 1 min, washed several times with SDW, and blotted dry with Kimwipe paper towels. The stems were cut into transverse slices manually with a scalpel. Stem slices were placed on a PDA medium and incubated at 28°C. After 3 days, the observed fungal mycelium was transferred to new Petri dishes to obtain axenic cultures.

The Qiagen plant DNA extraction kit (Hilden, Germany) was used to extract the DNA of three different fungal colonies isolated from the tomato stems. Fol4287 strain-specific genes FOXG_22560 and FOXG_18682 were amplified by PCR using the primers: Fol4287_22560_For: ATGCGCTTC AATGTTCTCGC and Fol4287_22560_Rev: ACAACAGACAGTACCAGCGG, as well as Fol4287_18682_For: GCTGCTACGGCGATACTGTC and Fol4287_18682_Rev: GACTCGTCTGGGCTGTACTC with 63°C and 64°C of alignment temperature respectively, 35 cycles and following the manufacturer instructions of Phusion High-Fidelity DNA Polymerase (Thermo Scientific). Finally, the PCR products were visualized in 1.2% agarose gels.

### Contour-CHEF electrophoresis

The harvested conidia sample was washed with sterile water, followed by 1.2 M KCl, resuspended in protoplasting solution (25 mg/mL driselase, 5 mg/mL lysing enzyme from Sigma-Aldrich, and 1.2 M KCl), and incubated overnight at 30°C under shaking at 80 rpm. The protoplasts were centrifuged at 4°C for 15 min at 1,000 g, washed by slowly resuspending in 10 mL STC buffer (1 M sorbitol, 10 mM Tris-HCl [pH 7.4], and 10 mM CaCl_2_), and centrifuged again as above. The supernatant was carefully poured out, and the protoplasts were resuspended in STE buffer (2% [wt/vol] SDS, 0.5 M Tris-HCl [pH 8.1], and 10 mM EDTA) to a titer of 2 × 10^8^ conidia/mL. The protoplasts were incubated at 50°C for 10 min and mixed at a 1:1 (vol/vol) ratio with 1.2% (wt/vol) Bio-Rad Certified Low Melt Agarose. The mixture was transferred to CHEF Disposable Plug Molds and stored at 4°C for long-term storage. CHEF gel electrophoresis was performed as previously described (77). Briefly, the CHEF Mapper System (Bio-Rad) using 1% (wt/vol) SeaKem Gold Agarose with optimized pulse parameters for separation of chromosomes in the 0.5-3.5 Mb range in 0.5× Tris borate EDTA (TBE) buffer at 4–7°C for 260 h. The switching time was 1,200–4,800 s at 1.5 V/cm, and the angle was 120°. The running buffer was changed every 2–3 days. The gels were stained with 3× GelGreen (Biotium).

### Genome alignment

The alignments of the Fol4287 and MRL8996 nuclear and mitochondrial genomes with repeats masked were generated using MUMmer version 3.23 (78) with the option “nucmer --maxmatch.” Alignments less than 1 kb were removed using “delta-filter -g -l 1000.” The extent of sequence identity was calculated for each chromosome by multiplying the percentage sequence identity for each alignment by the length of the alignment averaged over the total length of alignments for the chromosome. The circular plot was generated using Circos (79). Only alignments longer than 5 kb and contigs longer than 5 kb were considered. Contigs from the same chromosome were concatenated. A sequence alignment plot for the mitochondrial genome was generated via a PlotMUMmerAlignments.m script (available at https://github.com/d-ayhan/tools). The annotation of the Fol4287 genome was obtained from the 2010 assembly, while the MRL8996 genome was annotated *de novo* at JGI (55, 80). The genes in ACs were identified, and their enriched GO terms were analyzed. For Fol4287 genes, locus tag IDs starting with “FOXG_” were used, while for MRL8996, the “protein_id” numbers assigned by JGI were used.

### Repeat analysis

Repeats were *de novo* identified and classified using RepeatModeler v1.0.11 (81). A previously curated library of 69 TEs was included in the repeat sequence database. Usearch with the option “-id 0.75” was used to cluster the sequences around centroid repeat sequences, and the annotations were fixed manually (82). RepeatMasker version 4.0.7 was used to mask and annotate the genome assemblies (83). TECNEstimator, a bioinformatics pipeline used to quantify the copy numbers of repeat sequences using short reads from whole-genome sequencing data (SRA accessions: SRP140697 and SRP214968), was used to estimate read counts (available at https://github.com/d-ayhan/TECNEstimator). The counts were normalized to the median read coverages of the samples. For both genomes, only one representative sequence in a cluster with the highest copy number was selected for downstream analysis.

### GO term enrichment analysis

The genes harbored by ACs were identified as those located in AC contigs of MRL8996 and AC and unmapped contigs of Fol4287. The orthologous genes were identified using OrthoFinder version 2.3.3 with default options (84). GO terms for the Fol4287 and MRL8996 genomes were downloaded from JGI Mycocosm (JGI-specific genome identifiers: Fusox2 and FoxMRL8996, respectively) (55). The GO_term_enrichment_analysis.m script (available at https://github.com/d-ayhan/tools), which utilizes the hypergeometric cumulative distribution function, was used to analyze the enriched GO terms in ACs. The proteins encoded by the genes included in each term were subjected to conserved domain analysis using an InterPro-based annotated file provided by JGI and a NCBI conserved domain database CD-Search (https://www.ncbi.nlm.nih.gov/Structure/cdd/wrpsb.cgi).

### Phylogenetic analysis

The protein sequence alignments were generated by MEGA11 (85) using the ClustalW algorithm. The phylogenetic tree was reconstructed using FastTree (86) with default options and visualized in iTOL (87).

## SUPPLEMENTAL MATERIAL

File S1 mbio.00951-25-s0001.txt Fol4287 TEs sequences in fasta format.

File S2 mbio.00951-25-s0002.txt MRL8996 TEs sequences in fasta format.

Supplemental figures mbio.00951-25-s0003.pdf Fig. S1-S6.

Supplemental tables mbio.00951-25-s0004.xlsx Tables S1 to S6.

ASM does not own the copyrights to Supplemental Material that may be linked to, or accessed through, an article. The authors have granted ASM a non-exclusive, world-wide license to publish the Supplemental Material files. Please contact the corresponding author directly for reuse.

## References

[1] World Health Organization. 2022. WHO fungal priority pathogens list to guide research, development and public health action. Available from: https://www.who.int/publications-detail-redirect/9789240060241

[2] Dean R, Van Kan JAL, Pretorius ZA, Hammond-Kosack KE, Di Pietro A, Spanu PD, Rudd JJ, Dickman M, Kahmann R, Ellis J, Foster GD. 2012. The Top 10 fungal pathogens in molecular plant pathology. Mol Plant Pathol 13:414–430. doi:10.1111/j.1364-3703.2011.00783.x 22471698 PMC6638784

[3] Kredics L, Narendran V, Shobana CS, Vágvölgyi C, Manikandan P, Indo-Hungarian Fungal Keratitis Working Group. 2015. Filamentous fungal infections of the cornea: a global overview of epidemiology and drug sensitivity. Mycoses 58:243–260. doi:10.1111/myc.12306 25728367

[4] Abbondante S, Leal SM, Clark HL, Ratitong B, Sun Y, Ma L-J, Pearlman E. 2023. Immunity to pathogenic fungi in the eye. Semin Immunol 67:101753. doi:10.1016/j.smim.2023.101753 37060806 PMC10508057

[5] Hassan AS, Al-Hatmi AMS, Shobana CS, van Diepeningen AD, Kredics L, Vágvölgyi C, Homa M, Meis JF, de Hoog GS, Narendran V, Manikandan P, IHFK Working Group. 2016. Antifungal susceptibility and phylogeny of opportunistic members of the genus fusarium causing human keratomycosis in South India. Med Mycol 54:287–294. doi:10.1093/mmy/myv105 26705832

[6] Lalitha P, Prajna NV, Manoharan G, Srinivasan M, Mascarenhas J, Das M, D’Silva SS, Porco TC, Keenan JD. 2015. Trends in bacterial and fungal keratitis in South India, 2002-2012. Br J Ophthalmol 99:192–194. doi:10.1136/bjophthalmol-2014-305000 25143391 PMC7325420

[7] He D, Hao J, Zhang B, Yang Y, Song W, Zhang Y, Yokoyama K, Wang L. 2011. Pathogenic spectrum of fungal keratitis and specific identification of Fusarium solani. Invest Ophthalmol Vis Sci 52:2804–2808. doi:10.1167/iovs.10-5977 21273551

[8] Wang L, Sun S, Jing Y, Han L, Zhang H, Yue J. 2009. Spectrum of fungal keratitis in central China. Clin Exp Ophthalmol 37:763–771. doi:10.1111/j.1442-9071.2009.02155.x 19878220

[9] O’Sullivan J, Gilbert C, Foster A. 1997. The causes of childhood blindness in South Africa. S Afr Med J 87:1691–1695. 9497836

[10] Ibrahim MM, Vanini R, Ibrahim FM, Fioriti LS, Furlan EMR, Provinzano LMA, De Castro RS, Sousa SJDFE, Rocha EM. 2009. Epidemiologic aspects and clinical outcome of fungal keratitis in southeastern Brazil. Eur J Ophthalmol 19:355–361. doi:10.1177/112067210901900305 19396778

[11] O’Donnell K, Sarver BAJ, Brandt M, Chang DC, Noble-Wang J, Park BJ, Sutton DA, Benjamin L, Lindsley M, Padhye A, Geiser DM, Ward TJ. 2007. Phylogenetic diversity and microsphere array-based genotyping of human pathogenic Fusaria, including isolates from the multistate contact lens-associated U.S. keratitis outbreaks of 2005 and 2006. J Clin Microbiol 45:2235–2248. doi:10.1128/JCM.00533-07 17507522 PMC1933018

[12] Gower EW, Keay LJ, Oechsler RA, Iovieno A, Alfonso EC, Jones DB, Colby K, Tuli SS, Patel SR, Lee SM, Irvine J, Stulting RD, Mauger TF, Schein OD. 2010. Trends in fungal keratitis in the United States, 2001 to 2007. Ophthalmology 117:2263–2267. doi:10.1016/j.ophtha.2010.03.048 20591493

[13] Michielse CB, Rep M. 2009. Pathogen profile update: Fusarium oxysporum. Mol Plant Pathol 10:311–324. doi:10.1111/j.1364-3703.2009.00538.x 19400835 PMC6640313

[14] Berger S, El Chazli Y, Babu AF, Coste AT. 2017. Azole resistance in Aspergillus fumigatus: a consequence of antifungal use in agriculture? Front Microbiol 8:1024. doi:10.3389/fmicb.2017.01024 28638374 PMC5461301

[15] Pfaller MA. 2012. Antifungal drug resistance: mechanisms, epidemiology, and consequences for treatment. Am J Med 125:S3–13. doi:10.1016/j.amjmed.2011.11.001 22196207

[16] Altus V, Li-Jun M, Augusta M. 2020. *Fusarium* wilt (Panama disease) and monoculture in banana production: resurgence of a century-old disease, p 159–184. In Emerging plant diseases and global food security. A. Records & J. Ristaino, APS Press.

[17] Scheel CM, Hurst SF, Barreiros G, Akiti T, Nucci M, Balajee SA. 2013. Molecular analyses of Fusarium isolates recovered from a cluster of invasive mold infections in a Brazilian hospital. BMC Infect Dis 13:49. doi:10.1186/1471-2334-13-49 23363475 PMC3579725

[18] Nucci M, Marr KA, Vehreschild MJGT, de Souza CA, Velasco E, Cappellano P, Carlesse F, Queiroz-Telles F, Sheppard DC, Kindo A, et al.. 2014. Improvement in the outcome of invasive fusariosis in the last decade. Clin Microbiol Infect 20:580–585. doi:10.1111/1469-0691.12409 24118322

[19] Prajna NV, Krishnan T, Rajaraman R, Patel S, Srinivasan M, Das M, Ray KJ, O’Brien KS, Oldenburg CE, McLeod SD, Zegans ME, Porco TC, Acharya NR, Lietman TM, Rose-Nussbaumer J, Mycotic Ulcer Treatment Trial II Group. 2016. Effect of oral voriconazole on fungal keratitis in the mycotic ulcer treatment Trial II (MUTT II): a randomized clinical trial. JAMA Ophthalmol 134:1365–1372. doi:10.1001/jamaophthalmol.2016.4096 27787540 PMC6044431

[20] Kistler H. 2001. Evolution of host specificity in *Fusarium oxysporum*, p 70–82. In Nelson P, Summerell B (ed), Fusarium: Paul E. Nelson memorial symposium. APS Press.

[21] Ma L-J, van der Does HC, Borkovich KA, Coleman JJ, Daboussi M-J, Di Pietro A, Dufresne M, Freitag M, Grabherr M, Henrissat B, et al.. 2010. Comparative genomics reveals mobile pathogenicity chromosomes in Fusarium. Nature 464:367–373. doi:10.1038/nature08850 20237561 PMC3048781

[22] Ma L-J, Geiser DM, Proctor RH, Rooney AP, O’Donnell K, Trail F, Gardiner DM, Manners JM, Kazan K. 2013. Fusarium pathogenomics. Annu Rev Microbiol 67:399–416. doi:10.1146/annurev-micro-092412-155650 24024636

[23] van Dam P, Fokkens L, Schmidt SM, Linmans JHJ, Kistler HC, Ma L-J, Rep M. 2016. Effector profiles distinguish formae speciales of Fusarium oxysporum. Environ Microbiol 18:4087–4102. doi:10.1111/1462-2920.13445 27387256

[24] Yu H, Ayhan DH, Martínez-Soto D, Cochavi SM, Ma L-J. 2023. Accessory chromosomes of the *Fusarium oxysporum* species complex and their contribution to host niche adaptation, p 371–388. In Scott B, Mesarich C (ed), Plant relationships. Springer International Publishing, Cham.

[25] Rep M. 2005. Small proteins of plant-pathogenic fungi secreted during host colonization. FEMS Microbiol Lett 253:19–27. doi:10.1016/j.femsle.2005.09.014 16216445

[26] Yu H, Yang H, Haridas S, Hayes RD, Lynch H, Andersen S, Newman M, Li G, Martínez-Soto D, Milo-Cochavi S, Hazal Ayhan D, Zhang Y, Grigoriev IV, Ma L-J. 2023. Conservation and expansion of transcriptional factor repertoire in the Fusarium oxysporum species complex. J Fungi (Basel) 9:359. doi:10.3390/jof9030359 36983527 PMC10056406

[27] Turrà D, Segorbe D, Di Pietro A. 2014. Protein kinases in plant-pathogenic fungi: conserved regulators of infection. Annu Rev Phytopathol 52:267–288. doi:10.1146/annurev-phyto-102313-050143 25090477

[28] López-Berges MS, Rispail N, Prados-Rosales RC, Di Pietro A. 2010. A nitrogen response pathway regulates virulence functions in Fusarium oxysporum via the protein kinase TOR and the bZIP protein MeaB. Plant Cell 22:2459–2475. doi:10.1105/tpc.110.075937 20639450 PMC2929112

[29] Kistler HC, Rep M, Ma L-J. 2013. Structural dynamics of *Fusarium* genomes. In Brown DW, Proctor RH (ed), Fusarium: genomics, molecular and cellular biology. Horizon Scientific Press.

[30] van Dam P, Fokkens L, Ayukawa Y, van der Gragt M, Ter Horst A, Brankovics B, Houterman PM, Arie T, Rep M. 2017. A mobile pathogenicity chromosome in Fusarium oxysporum for infection of multiple cucurbit species. Sci Rep 7:9042. doi:10.1038/s41598-017-07995-y 28831051 PMC5567276

[31] Schäfer K, Di Pietro A, Gow NAR, MacCallum D. 2014. Murine model for Fusarium oxysporum invasive fusariosis reveals organ-specific structures for dissemination and long-term persistence. PLoS ONE 9:e89920. doi:10.1371/journal.pone.0089920 24587124 PMC3937399

[32] Zhang Y, Yang H, Turra D, Zhou S, Ayhan DH, DeIulio GA, Guo L, Broz K, Wiederhold N, Coleman JJ, Donnell KO, Youngster I, McAdam AJ, Savinov S, Shea T, Young S, Zeng Q, Rep M, Pearlman E, Schwartz DC, Di Pietro A, Kistler HC, Ma L-J. 2020. The genome of opportunistic fungal pathogen Fusarium oxysporum carries a unique set of lineage-specific chromosomes. Commun Biol 3:1–12. doi:10.1038/s42003-020-0770-2 32005944 PMC6994591

[33] Schmidt SM, Houterman PM, Schreiver I, Ma L, Amyotte S, Chellappan B, Boeren S, Takken FLW, Rep M. 2013. MITEs in the promoters of effector genes allow prediction of novel virulence genes in Fusarium oxysporum. BMC Genomics 14:119. doi:10.1186/1471-2164-14-119 23432788 PMC3599309

[34] Chang DC, Grant GB, O’Donnell K, Wannemuehler KA, Noble-Wang J, Rao CY, Jacobson LM, Crowell CS, Sneed RS, Lewis FMT, Schaffzin JK, Kainer MA, Genese CA, Alfonso EC, Jones DB, Srinivasan A, Fridkin SK, Park BJ, Fusarium Keratitis Investigation Team. 2006. Multistate outbreak of Fusarium keratitis associated with use of a contact lens solution. JAMA 296:953–963. doi:10.1001/jama.296.8.953 16926355

[35] Leal SM, Vareechon C, Cowden S, Cobb BA, Latgé J-P, Momany M, Pearlman E. 2012. Fungal antioxidant pathways promote survival against neutrophils during infection. J Clin Invest 122:2482–2498. doi:10.1172/JCI63239 22706306 PMC3534057

[36] O’Donnell K, Sutton DA, Rinaldi MG, Magnon KC, Cox PA, Revankar SG, Sanche S, Geiser DM, Juba JH, van Burik J-AH, Padhye A, Anaissie EJ, Francesconi A, Walsh TJ, Robinson JS. 2004 a. Genetic diversity of human pathogenic members of the Fusarium oxysporum complex inferred from multilocus DNA sequence data and amplified fragment length polymorphism analyses: evidence for the recent dispersion of a geographically widespread clonal lineage and nosocomial origin. J Clin Microbiol 42:5109–5120. doi:10.1128/JCM.42.11.5109-5120.2004 15528703 PMC525153

[37] Di Pietro A, Roncero MI. 1996. Purification and characterization of an exo-polygalacturonase from the tomato vascular wilt pathogen Fusarium oxysporum f.sp. lycopersici. FEMS Microbiol Lett 145:295–299. doi:10.1111/j.1574-6968.1996.tb08592.x 8961570

[38] Beisel KW, Hazlett LD, Berk RS. 1983. Dominant susceptibility effect on the murine corneal response to Pseudomonas aeruginosa. Exp Biol Med (Maywood) 172:488–491. doi:10.3181/00379727-172-41592 6844357

[39] Christensen JE, Andreasen SO, Christensen JP, Thomsen AR. 2001. CD11b expression as a marker to distinguish between recently activated effector CD8(+) T cells and memory cells. Int Immunol 13:593–600. doi:10.1093/intimm/13.4.593 11282998

[40] Di Pietro A, Roncero MI. 1998. Cloning, expression, and role in pathogenicity of pg1 encoding the major extracellular endopolygalacturonase of the vascular wilt pathogen Fusarium oxysporum. Mol Plant Microbe Interact 11:91–98. doi:10.1094/MPMI.1998.11.2.91 9450333

[41] Guo L, Yu H, Wang B, Vescio K, DeIulio GA, Yang H, Berg A, Zhang L, Edel-Hermann V, Steinberg C, Kistler HC, Ma L-J. 2021. Metatranscriptomic comparison of endophytic and pathogenic Fusarium –Arabidopsis interactions reveals plant transcriptional plasticity. MPMI 34:1071–1083. doi:10.1094/MPMI-03-21-0063-R 33856230 PMC9048145

[42] Martínez-Soto D, Yu H, Allen KS, Ma L-J. 2023. Differential colonization of the plant vasculature between endophytic versus pathogenic Fusarium oxysporum strains. Mol Plant Microbe Interact 36:4–13. doi:10.1094/MPMI-08-22-0166-SC 36279112 PMC10052776

[43] García-Maceira FI, Di Pietro A, Roncero MI. 2000. Cloning and disruption of pgx4 encoding an in planta expressed exopolygalacturonase from Fusarium oxysporum. Mol Plant Microbe Interact 13:359–365. doi:10.1094/MPMI.2000.13.4.359 10755298

[44] Ghareeb H, Becker A, Iven T, Feussner I, Schirawski J. 2011. Sporisorium reilianum infection changes inflorescence and branching architectures of maize. Plant Physiol 156:2037–2052. doi:10.1104/pp.111.179499 21653782 PMC3149921

[45] Kessel L, Johnson L, Arvidsson H, Larsen M. 2010. The relationship between body and ambient temperature and corneal temperature. Invest Ophthalmol Vis Sci 51:6593. doi:10.1167/iovs.10-5659 20671277

[46] Pfaller MA, Diekema DJ, Messer SA, Hollis RJ, Jones RN. 2003. In vitro activities of caspofungin compared with those of fluconazole and itraconazole against 3,959 clinical isolates of Candida spp., including 157 fluconazole-resistant isolates. Antimicrob Agents Chemother 47:1068–1071. doi:10.1128/AAC.47.3.1068-1071.2003 12604543 PMC149290

[47] Bowman JC, Hicks PS, Kurtz MB, Rosen H, Schmatz DM, Liberator PA, Douglas CM. 2002. The antifungal echinocandin caspofungin acetate kills growing cells of Aspergillus fumigatus in vitro. Antimicrob Agents Chemother 46:3001–3012. doi:10.1128/AAC.46.9.3001-3012.2002 12183260 PMC127409

[48] Ha Y, Covert SF, Momany M. 2006. FsFKS1, the 1,3-beta-glucan synthase from the caspofungin-resistant fungus Fusarium solani. Eukaryot Cell 5:1036–1042. doi:10.1128/EC.00030-06 16835448 PMC1489279

[49] Pfaller MA, Marco F, Messer SA, Jones RN. 1998. In vitro activity of two echinocandin derivatives, LY303366 and MK-0991 (L-743,792), against clinical isolates of Aspergillus, Fusarium, Rhizopus, and other filamentous fungi. Diagn Microbiol Infect Dis 30:251–255. doi:10.1016/S0732-8893(97)00246-0 9582584

[50] Ayhan DH, López-Díaz C, Di Pietro A, Ma L-J. 2018. Improved assembly of reference genome Fusarium oxysporum f. sp. lycopersici strain Fol4287. Microbiol Resour Announc 7:e00910-18. doi:10.1128/MRA.00910-18 30533622 PMC6256600

[51] Fokkens L, Shahi S, Connolly LR, Stam R, Schmidt SM, Smith KM, Freitag M, Rep M. 2018. The multi-speed genome of Fusarium oxysporum reveals association of histone modifications with sequence divergence and footprints of past horizontal chromosome transfer events. Genomics. doi:10.1101/465070

[52] Brankovics B, van Dam P, Rep M, de Hoog GS, J van der Lee TA, Waalwijk C, van Diepeningen AD. 2017. Mitochondrial genomes reveal recombination in the presumed asexual Fusarium oxysporum species complex. BMC Genomics 18:735. doi:10.1186/s12864-017-4116-5 28923029 PMC5604515

[53] Yang H, Yu H, Ma LJ. 2020. Accessory chromosomes in Fusarium oxysporum. Phytopathology 110:1488–1496. doi:10.1094/PHYTO-03-20-0069-IA 32692281 PMC8086798

[54] Chellapan BV, van Dam P, Rep M, Cornelissen BJC, Fokkens L. 2016. Non-canonical helitrons in Fusarium oxysporum. Mob DNA 7:27. doi:10.1186/s13100-016-0083-7 27990178 PMC5148889

[55] Grigoriev IV, Nikitin R, Haridas S, Kuo A, Ohm R, Otillar R, Riley R, Salamov A, Zhao X, Korzeniewski F, Smirnova T, Nordberg H, Dubchak I, Shabalov I. 2014. MycoCosm portal: gearing up for 1000 fungal genomes. Nucleic Acids Res 42:D699–D704. doi:10.1093/nar/gkt1183 24297253 PMC3965089

[56] Lachner M, O’Carroll D, Rea S, Mechtler K, Jenuwein T. 2001. Methylation of histone H3 lysine 9 creates a binding site for HP1 proteins. Nature 410:116–120. doi:10.1038/35065132 11242053

[57] Nakayama J, Rice JC, Strahl BD, Allis CD, Grewal SIS. 2001. Role of histone H3 lysine 9 methylation in epigenetic control of heterochromatin assembly. Science 292:110–113. doi:10.1126/science.1060118 11283354

[58] Mondal T, Rasmussen M, Pandey GK, Isaksson A, Kanduri C. 2010. Characterization of the RNA content of chromatin. Genome Res 20:899–907. doi:10.1101/gr.103473.109 20404130 PMC2892091

[59] Varadi M, Anyango S, Deshpande M, Nair S, Natassia C, Yordanova G, Yuan D, Stroe O, Wood G, Laydon A, et al.. 2022. AlphaFold Protein Structure Database: massively expanding the structural coverage of protein-sequence space with high-accuracy models. Nucleic Acids Res 50:D439–D444. doi:10.1093/nar/gkab1061 34791371 PMC8728224

[60] Leopold K, Stirpe A, Schalch T. 2019. Transcriptional gene silencing requires dedicated interaction between HP1 protein Chp2 and chromatin remodeler Mit1. Genes Dev 33:565–577. doi:10.1101/gad.320440.118 30808655 PMC6499331

[61] Schoelz JM, Riddle NC. 2022. Functions of HP1 proteins in transcriptional regulation. Epigenetics Chromatin 15:14. doi:10.1186/s13072-022-00453-8 35526078 PMC9078007

[62] DeIulio GA, Guo L, Zhang Y, Goldberg JM, Kistler HC, Ma L-J. 2018. Kinome expansion in the Fusarium oxysporum species complex driven by accessory chromosomes. mSphere 3:e00231-18. doi:10.1128/mSphere.00231-18 29898984 PMC6001611

[63] van der Does HC, Duyvesteijn RGE, Goltstein PM, van Schie CCN, Manders EMM, Cornelissen BJC, Rep M. 2008. Expression of effector gene SIX1 of Fusarium oxysporum requires living plant cells. Fungal Genet Biol 45:1257–1264. doi:10.1016/j.fgb.2008.06.002 18606236

[64] Lievens B, Houterman PM, Rep M. 2009. Effector gene screening allows unambiguous identification of Fusarium oxysporum f. sp. lycopersici races and discrimination from other formae speciales. FEMS Microbiol Lett 300:201–215. doi:10.1111/j.1574-6968.2009.01783.x 19799634

[65] Houterman PM, Ma L, van Ooijen G, de Vroomen MJ, Cornelissen BJC, Takken FLW, Rep M. 2009. The effector protein Avr2 of the xylem-colonizing fungus Fusarium oxysporum activates the tomato resistance protein I-2 intracellularly. Plant J 58:970–978. doi:10.1111/j.1365-313X.2009.03838.x 19228334

[66] Lamb C, Dixon RA. 1997. The oxidative burst in plant disease resistance. Annu Rev Plant Physiol Plant Mol Biol 48:251–275. doi:10.1146/annurev.arplant.48.1.251 15012264

[67] Segal LM, Wilson RA. 2018. Reactive oxygen species metabolism and plant-fungal interactions. Fungal Genet Biol 110:1–9. doi:10.1016/j.fgb.2017.12.003 29225185

[68] Valero C, Colabardini AC, de Castro PA, Amich J, Bromley MJ, Goldman GH. 2022. The caspofungin paradoxical effect is a tolerant “Eagle effect” in the filamentous fungal pathogen Aspergillus fumigatus. MBio 13:e0044722. doi:10.1128/mbio.00447-22 35420487 PMC9239232

[69] Sumiyoshi M, Miyazaki T, Makau JN, Mizuta S, Tanaka Y, Ishikawa T, Makimura K, Hirayama T, Takazono T, Saijo T, Yamaguchi H, Shimamura S, Yamamoto K, Imamura Y, Sakamoto N, Obase Y, Izumikawa K, Yanagihara K, Kohno S, Mukae H. 2020. Novel and potent antimicrobial effects of caspofungin on drug-resistant Candida and bacteria. Sci Rep 10:17745. doi:10.1038/s41598-020-74749-8 33082485 PMC7576149

[70] Day AM, Quinn J. 2019. Stress-activated protein kinases in human fungal pathogens. Front Cell Infect Microbiol 9:261. doi:10.3389/fcimb.2019.00261 31380304 PMC6652806

[71] García R, Bravo E, Diez-Muñiz S, Nombela C, Rodríguez-Peña JM, Arroyo J. 2017. A novel connection between the Cell Wall Integrity and the PKA pathways regulates cell wall stress response in yeast. Sci Rep 7:5703. doi:10.1038/s41598-017-06001-9 28720901 PMC5515849

[72] Iyer KR, Robbins N, Cowen LE. 2022. The role of Candida albicans stress response pathways in antifungal tolerance and resistance. iScience 25:103953. doi:10.1016/j.isci.2022.103953 35281744 PMC8905312

[73] Pianalto KM, Billmyre RB, Telzrow CL, Alspaugh JA. 2019. Roles for stress response and cell wall biosynthesis pathways in caspofungin tolerance in Cryptococcus neoformans Genetics 213:213–227. doi:10.1534/genetics.119.302290 31266771 PMC6727808

[74] Ries LNA, Rocha MC, de Castro PA, Silva-Rocha R, Silva RN, Freitas FZ, de Assis LJ, Bertolini MC, Malavazi I, Goldman GH. 2017. The Aspergillus fumigatus CrzA transcription factor activates chitin synthase gene expression during the caspofungin paradoxical effect. MBio 8:e00705-17. doi:10.1128/mBio.00705-17 28611248 PMC5472186

[75] de Castro PA, Colabardini AC, Manfiolli AO, Chiaratto J, Silva LP, Mattos EC, Palmisano G, Almeida F, Persinoti GF, Ries LNA, Mellado L, Rocha MC, Bromley M, Silva RN, de Souza GS, Loures FV, Malavazi I, Brown NA, Goldman GH. 2019. Aspergillus fumigatus calcium-responsive transcription factors regulate cell wall architecture promoting stress tolerance, virulence and caspofungin resistance. PLoS Genet 15:e1008551. doi:10.1371/journal.pgen.1008551 31887136 PMC6948819

[76] Leal SM Jr, Roy S, Vareechon C, Carrion S deJesus, Clark H, Lopez-Berges MS, Di Pietro A, Schrettl M, Beckmann N, Redl B, Haas H, Pearlman E. 2013. Targeting iron acquisition blocks infection with the fungal pathogens Aspergillus fumigatus and Fusarium oxysporum. PLoS Pathog 9:e1003436. doi:10.1371/journal.ppat.1003436 23853581 PMC3708856

[77] Ayukawa Y, Asai S, Gan P, Tsushima A, Ichihashi Y, Shibata A, Komatsu K, Houterman PM, Rep M, Shirasu K, Arie T. 2021. A pair of effectors encoded on a conditionally dispensable chromosome of Fusarium oxysporum suppress host-specific immunity. Commun Biol 4:707. doi:10.1038/s42003-021-02245-4 34108627 PMC8190069

[78] Kurtz S, Phillippy A, Delcher AL, Smoot M, Shumway M, Antonescu C, Salzberg SL. 2004. Versatile and open software for comparing large genomes. Genome Biol 5:R12. doi:10.1186/gb-2004-5-2-r12 14759262 PMC395750

[79] Krzywinski M, Schein J, Birol I, Connors J, Gascoyne R, Horsman D, Jones SJ, Marra MA. 2009. Circos: an information aesthetic for comparative genomics. Genome Res 19:1639–1645. doi:10.1101/gr.092759.109 19541911 PMC2752132

[80] Grigoriev IV, Cullen D, Goodwin SB, Hibbett D, Jeffries TW, Kubicek CP, Kuske C, Magnuson JK, Martin F, Spatafora JW, Tsang A, Baker SE. 2011. Fueling the future with fungal genomics. Mycology 2:192–209. doi:10.1080/21501203.2011.584577

[81] Smit A, Hubley R. 2015. RepeatModeler Open-1.0. Available from: http://www.repeatmasker.org

[82] Edgar R. 2010. Usearch. Berkeley, CA (United States) Lawrence Berkeley National Lab. (LBNL)

[83] Smit A, Hubley R, Green P. 2015. RepeatMasker Open-4.0. http://www.repeatmasker.org.

[84] Emms DM, Kelly S. 2019. OrthoFinder: phylogenetic orthology inference for comparative genomics. Genome Biol 20:238. doi:10.1186/s13059-019-1832-y 31727128 PMC6857279

[85] Tamura K, Stecher G, Kumar S. 2021. MEGA11: molecular evolutionary genetics analysis version 11. Mol Biol Evol 38:3022–3027. doi:10.1093/molbev/msab120 33892491 PMC8233496

[86] Price MN, Dehal PS, Arkin AP. 2010. FastTree 2--approximately maximum-likelihood trees for large alignments. PLoS One 5:e9490. doi:10.1371/journal.pone.0009490 20224823 PMC2835736

[87] Letunic I, Bork P. 2021. Interactive tree of life (iTOL) v5: an online tool for phylogenetic tree display and annotation. Nucleic Acids Res 49:W293–W296. doi:10.1093/nar/gkab301 33885785 PMC8265157

