# Supplementary material for: Host-specific adaptation in Fusarium oxysporum correlates with distinct accessory chromosome content in human and plant pathogenic strains
Source: mBio. 2025 Jun 26;16(8):e00951-25. doi: 10.1128/mbio.00951-25 (PMC12345182; doi:10.1128/mbio.00951-25)
Supplement: Supplemental figures — Fig. S1-S6. [file mbio.00951-25-s0003.pdf]

Supplementary Figure S1

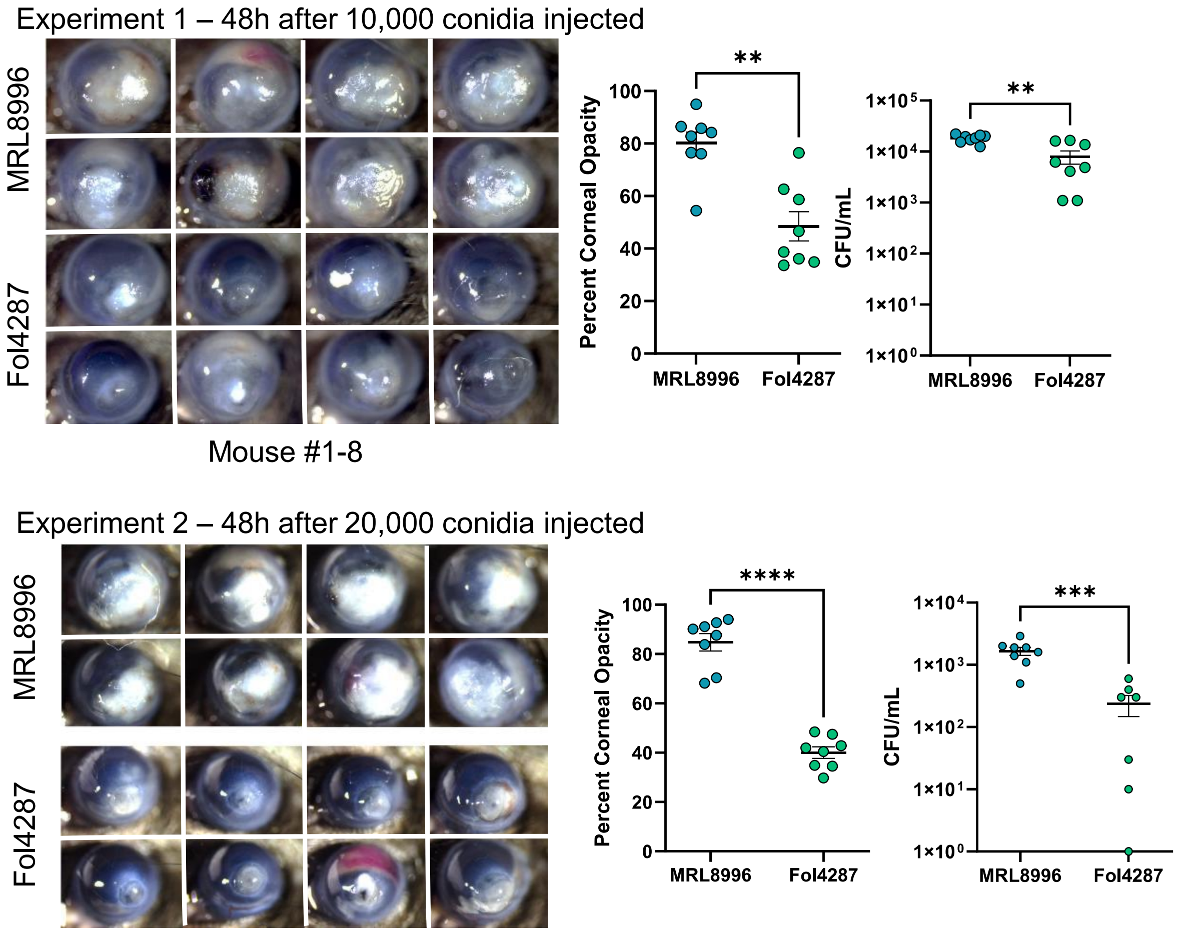

Figure S1. *In vivo* pathogenicity assay of clinical (MRL8996) and agricultural (Fol4287) *F. oxysporum* strains using the mouse keratitis model using different spore concentrations. Infected mouse corneas (n = 8) at 48 h after injecting swollen conidia directly into the corneal stroma. Viable conidia from infected corneas as indicated by the number of colony-forming units (CFUs) (** $p < 0.01$, *** $p < 0.001$). Significance was determined by a paired Student's *t*-test, where $p < 0.05$ was considered significant.

Supplementary Figure S2

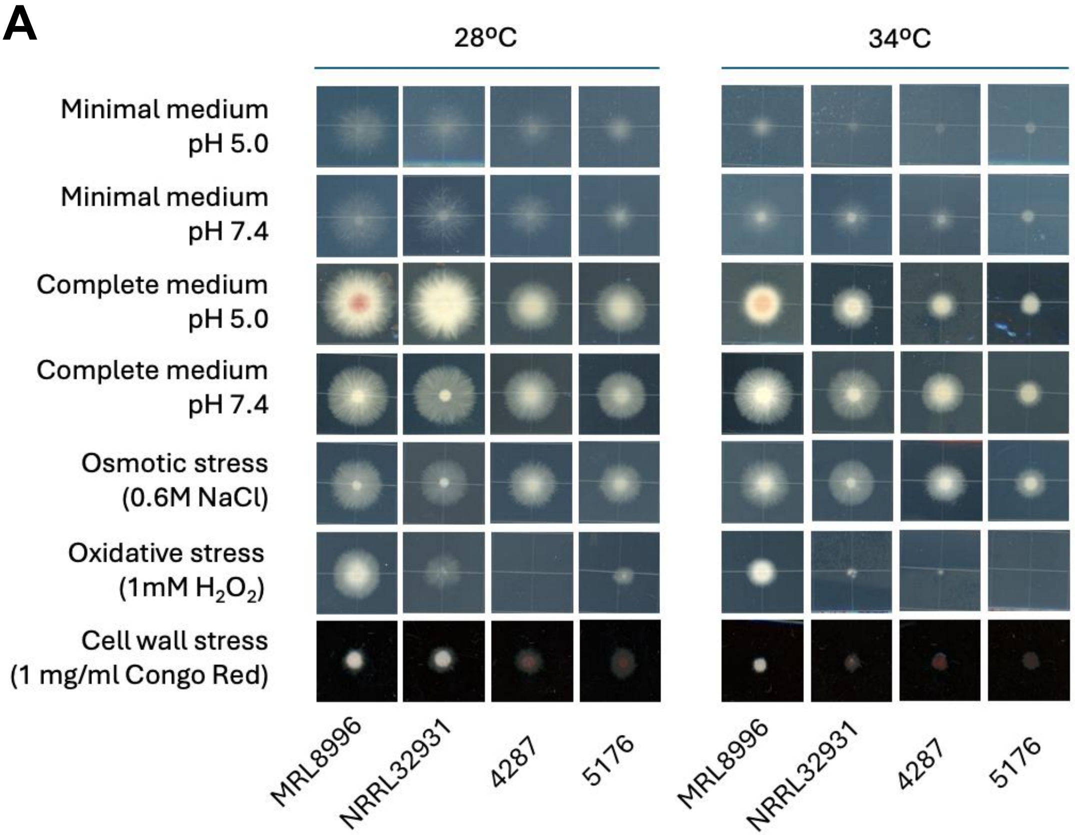

Supplementary Figure S2

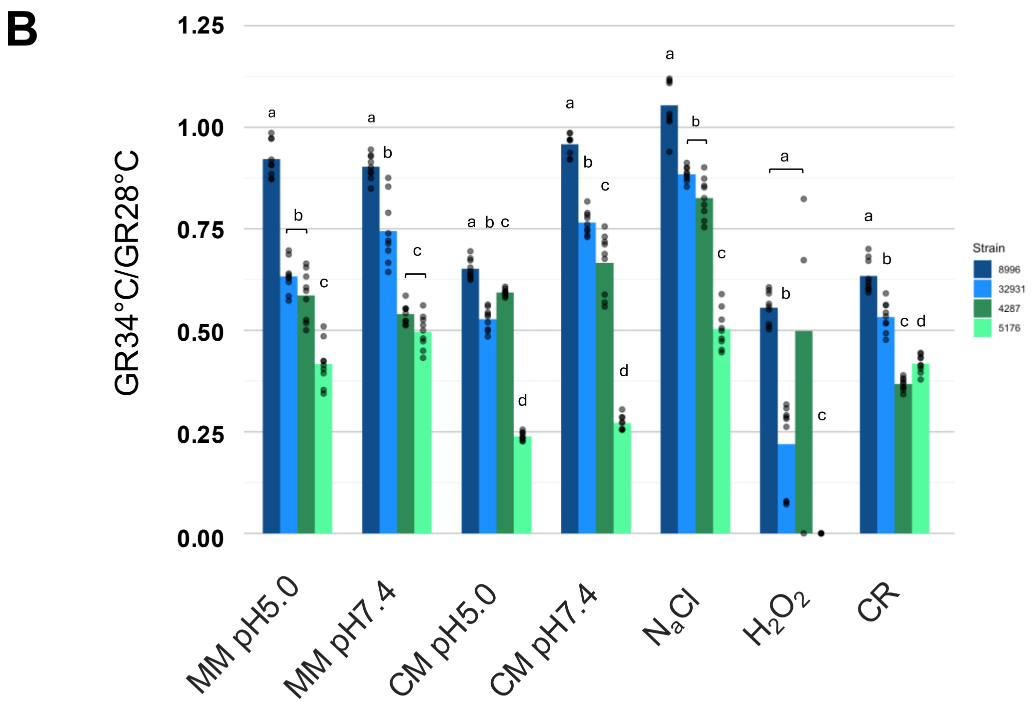

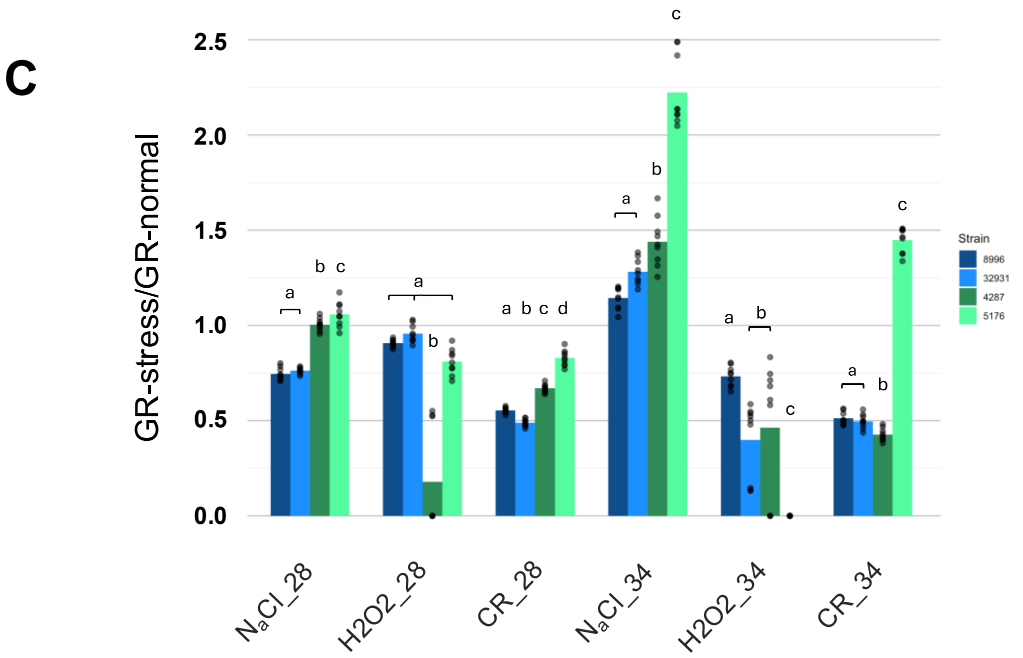

Supplementary Figure S2. ***In vitro*** **growth of clinical (MRL8996 and NRRL32931) and agricultural (Fol4287 and Fol5176)** ***F. oxysporum*** **strains subjected to abiotic stress.**

A. Colony morphology of MRL8996, NRRL32931, Fol4287 and Fol5176 on minimal medium pH ~5, minimal medium pH 7.4, complete medium pH ~5, complete medium pH 7.4, osmosis stress medium (PDA containing 0.6M NaCl), oxidative stress medium (PDA with 1mM H2O2), and cell wall stress medium (YPD with 1mg/ml Congo Red). The plates were incubated at 28ºC or 34ºC. The images are representative of three replicates and were taken at 2 days post-inoculation.

B. Ratio of the growth rates (GRs) of colonies under the same conditions as in at 34°C and 28°C (GR34°C/GR28°C) as a representation of temperature adaptation.

C. Ratio of mean rates of growth under stress conditions (GRstress) and in CM (GRnormal) as a representation of stress tolerance at 28°C and 34°C. Data points indicate each growth rate value at 34°C normalized to each growth rate value at 28°C. One way ANOVA test were performed within each condition across the four strains. The same letter represents no significant difference of variance.

Supplementary Figure S3

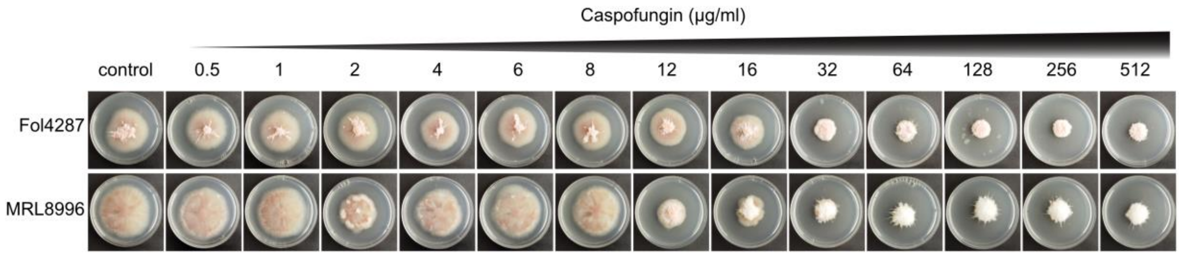

Figure S3 Both the keratitis (MRL8998) and plant (Fol4287) strains demonstrated enhanced tolerance to high concentrations of caspofungin in minimal media. Notably, the plant strain exhibited high resistance to caspofungin at concentrations up to 16 µg/mL. In contrast, the growth rate of the keratitis strain MRL8996 dropped by 50% under 2 µg/mL of caspofungin, then reached a 35% decrease at 8 µg/mL, relative to the control (Fig. 4B). This paradoxical caspofungin effect was also observed when measuring the time required to reach 50% conidial germination (Fig. 4C).

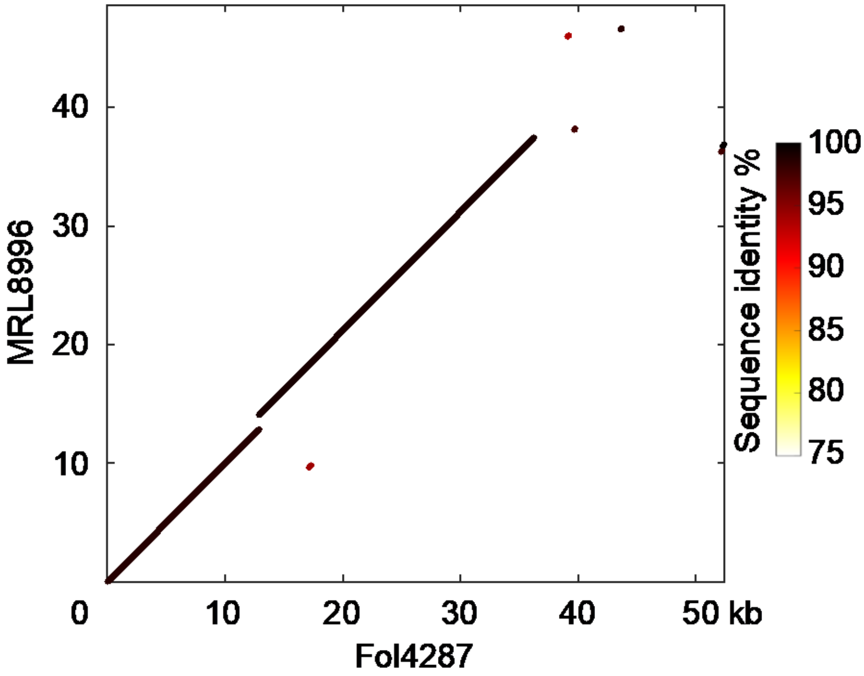

Figure S4 Overall conservation of the mitochondrial genomes in strain MRL 8996 and Fol4287 (99.0%). A divergent sequence around open reading frame 2285 (ORF2285) highlights a known variable mitochondrial region in the genus Fusarium.

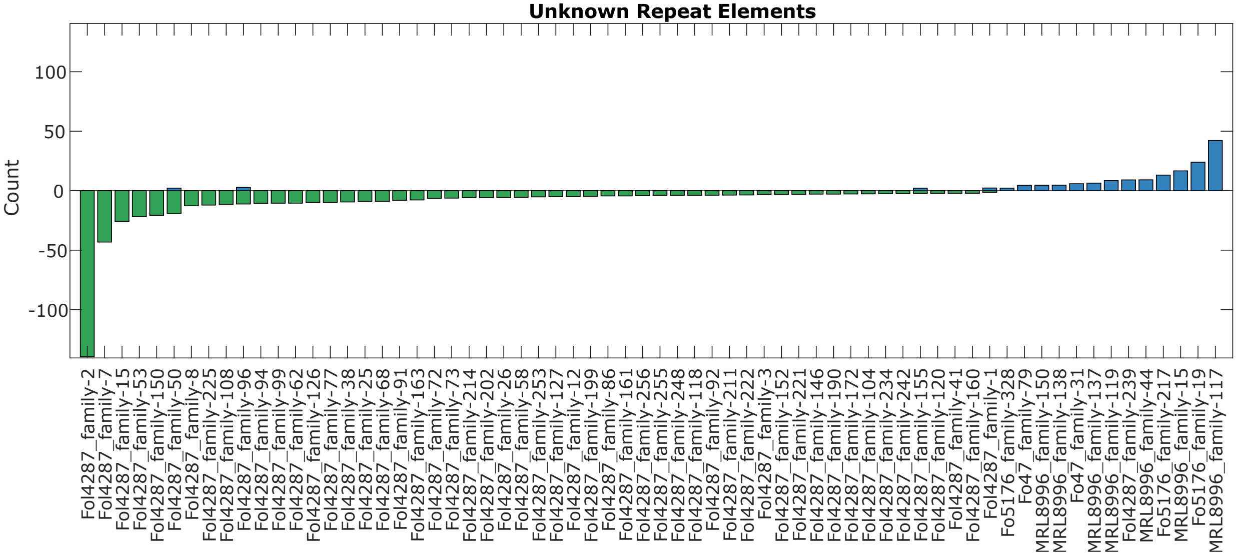

Figure S5. Distinct repeat contents of unknown repeat families in the Fol4287 (green) and MRL8996 (blue) genomes.

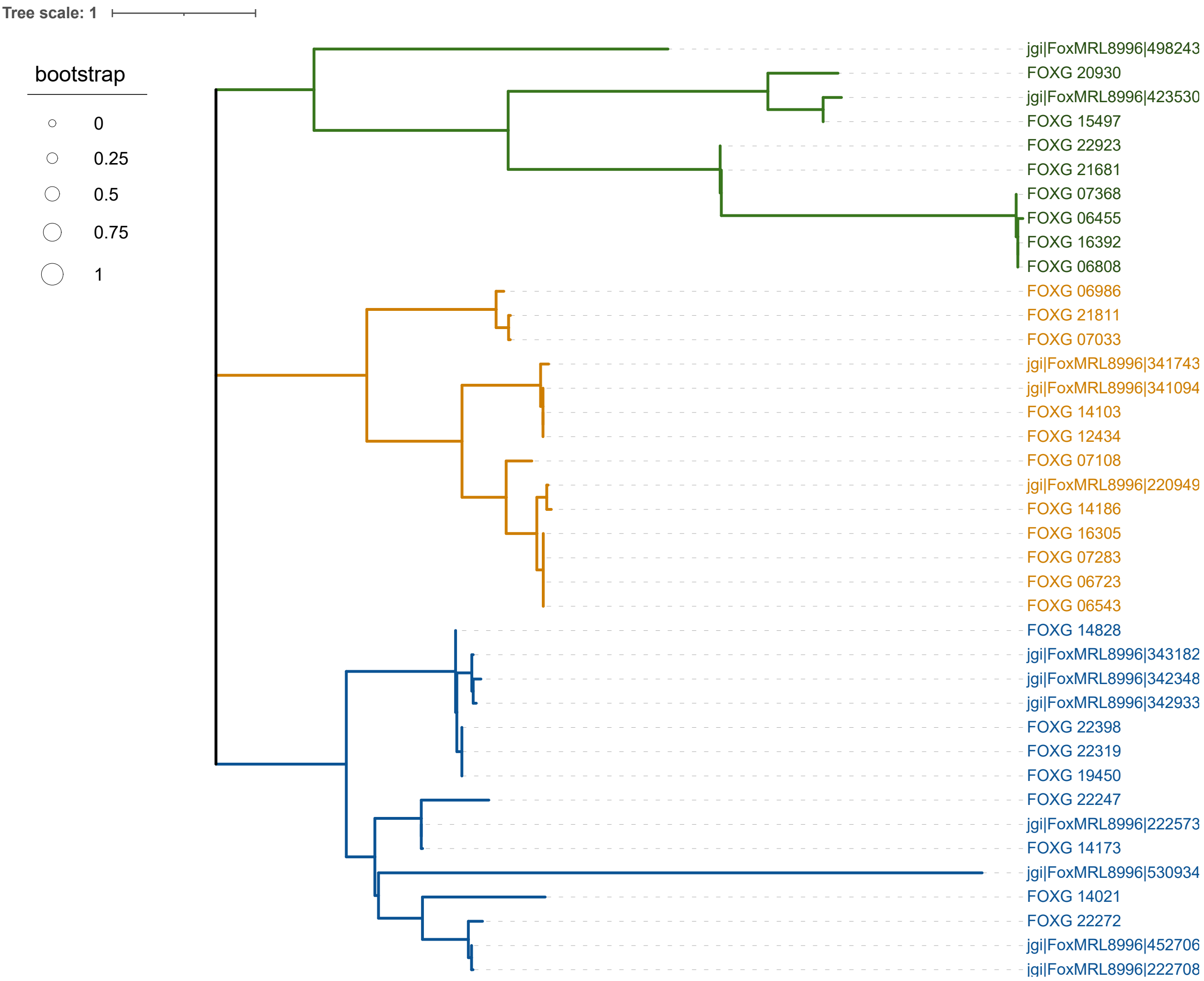

Figure S6. Phylogeny of proteins involved in chromatin assembly and disassembly encoded by genes located on the accessory chromosomes of Fol4287 and MRL8996. There are three clades highlighted in green, brown and blue colors. The first clade shares homologs of CHROMO domain-containing protein 2 (Chp2) from fission Schizosaccharomyces pombe. The second clade encodes homologs of putative Heterochromatin protein 1 (HP1) (A0A0C4BKY0) based on AlphaFold prediction. The third clade includes genes encoding Zinc finger C2H2-type domain-containing proteins.
